# Screening and Evaluation of New Hydroxymethylfurfural Oxidases for Furandicarboxylic Acid Production

**DOI:** 10.1128/AEM.00842-20

**Published:** 2020-08-03

**Authors:** Mario Viñambres, Marta Espada, Angel T. Martínez, Ana Serrano

**Affiliations:** a Centro de Investigaciones Biológicas Margarita Salas, CSIC, Madrid, Spain; North Carolina State University

**Keywords:** hydroxymethylfurfural, furandicarboxylic acid, database screening, *Pseudomonas* genes, *Escherichia coli* expression, flavooxidases, enzyme kinetics, enzyme stability, biotransformation, bioplastics

## Abstract

The enzymatic production of 2,5-furandicarboxylic acid (FDCA) from 5-hydroxymethylfurfural (HMF) has gained interest in recent years, as FDCA is a renewable precursor of poly(ethylene-2,5-furandicarboxylate) (PEF). 5-Hydroxymethylfurfural oxidases (HMFOs) form a flavoenzyme family with genes annotated in a dozen bacterial species but only one enzyme purified and characterized to date (after heterologous expression of a *Methylovorus* sp. HMFO gene). This oxidase acts on both furfuryl alcohols and aldehydes and, therefore, is able to catalyze the conversion of HMF into FDCA through 2,5-diformylfuran (DFF) and 2,5-formylfurancarboxylic acid (FFCA), with only the need of oxygen as a cosubstrate. To enlarge the repertoire of HMFO enzymes available, genetic databases were screened for putative HMFO genes, followed by heterologous expression in Escherichia coli. After unsuccessful trials with other bacterial HMFO genes, HMFOs from two *Pseudomonas* species were produced as active soluble enzymes, purified, and characterized. The *Methylovorus* sp. enzyme was also produced and purified in parallel for comparison. Enzyme stability against temperature, pH, and hydrogen peroxide, three key aspects for application, were evaluated (together with optimal conditions for activity), revealing differences between the three HMFOs. Also, the kinetic parameters for HMF, DFF, and FFCA oxidation were determined, the new HMFOs having higher efficiencies for the oxidation of FFCA, which constitutes the bottleneck in the enzymatic route for FDCA production. These results were used to set up the best conditions for FDCA production by each enzyme, attaining a compromise between optimal activity and half-life under different conditions of operation.

**IMPORTANCE** HMFO is the only enzyme described to date that can catalyze by itself the three consecutive oxidation steps to produce FDCA from HMF. Unfortunately, only one HMFO enzyme is currently available for biotechnological application. This availability is enlarged here by the identification, heterologous production, purification, and characterization of two new HMFOs, one from Pseudomonas nitroreducens and one from an unidentified *Pseudomonas* species. Compared to the previously known *Methylovorus* HMFO, the new enzyme from P. nitroreducens exhibits better performance for FDCA production in wider pH and temperature ranges, with higher tolerance for the hydrogen peroxide formed, longer half-life during oxidation, and higher yield and total turnover numbers in long-term conversions under optimized conditions. All these features are relevant properties for the industrial production of FDCA. In summary, gene screening and heterologous expression can facilitate the selection and improvement of HMFO enzymes as biocatalysts for the enzymatic synthesis of renewable building blocks in the production of bioplastics.

## Abstract (precis)

HMFO is the only enzyme described to date that can catalyze by itself the three consecutive oxidation steps to produce FDCA from HMF. Unfortunately, only one HMFO enzyme is currently available for biotechnological application. This availability is enlarged here by the identification, heterologous production, purification, and characterization of two new HMFOs, one from Pseudomonas nitroreducens and one from an unidentified *Pseudomonas* species. Compared to the previously known *Methylovorus* HMFO, the new enzyme from P. nitroreducens exhibits better performance for FDCA production in wider pH and temperature ranges, with higher tolerance for the hydrogen peroxide formed, longer half-life during oxidation, and higher yield and total turnover numbers in long-term conversions under optimized conditions. All these features are relevant properties for the industrial production of FDCA. In summary, gene screening and heterologous expression can facilitate the selection and improvement of HMFO enzymes as biocatalysts for the enzymatic synthesis of renewable building blocks in the production of bioplastics.

## INTRODUCTION

5-Hydroxymethylfurfural oxidases (HMFOs; EC 1.1.3.47) are flavoenzymes of biotechnological interest, classified in the superfamily of glucose-methanol-choline oxidases and dehydrogenases (GMC) (1). The importance of these enzymes resides in their ability to catalyze the three consecutive oxidation steps for the production of 2,5-furandicarboxylic acid (FDCA) from 5-hydroxymethylfurfural (HMF) with only the need of molecular oxygen as a cosubstrate. The final product, FDCA, is reported as a building block for the production of renewable and biodegradable plastics through its polymerization into poly(ethylene-2,5-furandicarboxylate) (PEF) (2), which is expected to substitute for petroleum-derived poly(ethylene-terephthalate) (PET) plastics in the near future.

HMF-oxidizing activity was first identified in Cupriavidus basilensis strain HMF14 (3, 4) and more recently in Pseudomonas putida strain ALS1267 (5), with their metabolic pathways being characterized. The *hmfH* gene of C. basilensis encodes an HMFO (UniProt D5KB61) that contributes to the conversion of HMF into FDCA, while the lack of this gene in P. putida suggests that, in this species, the conversion of HMF is catalyzed by different enzymes. The only HMFO characterized to date is that from *Methylovorus* sp. strain MP688 (*Metsp*HMFO), a homologue of the enzyme encoded by the *hmfH* gene of C. basilensis, which was produced in Escherichia coli (6). This flavoenzyme, with a flavin adenine dinucleotide (FAD) molecule as a prosthetic group, is active on primary alcohols, primary thiols, and hydrated aldehydes (6, 7). Its catalytic mechanism, similar to that of other GMC oxidases (8–11), involves a proton transfer from the hydroxyl (or thiol) group to a conserved catalytic base, *Metsp*HMFO His467 (12), and hydride abstraction from the substrate α-carbon by the oxidized flavin. The reduced flavin is then reoxidized by molecular oxygen, yielding hydrogen peroxide as a by-product (13).

**FIG 1:**
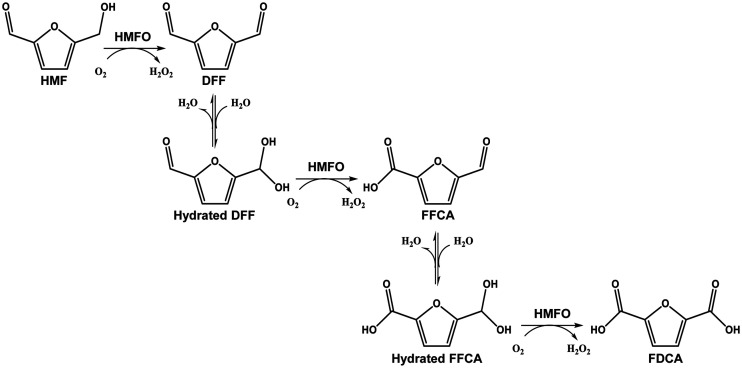
Reactions for conversion of HMF into FDCA by *Metsp*HMFO. First, the alcohol group of HMF is oxidized, resulting in DFF. Then, oxidation of the hydrated form (*gem*-diol) of DFF gives FFCA. Finally, hydrated FFCA is converted into FDCA.

The reported activity of HMFO on furfuryl alcohols and aldehydes makes this enzyme a suitable biocatalyst for the production of FDCA from HMF, which takes place through the hydrated 2,5-diformylfuran (DFF) and 2,5-formylfurancarboxylic acid (FFCA) *gem*-diol intermediates (Fig. 1) (14). Other oxidases, such as galactose oxidase (GAO) from Dactylium dendroides and glyoxal oxidase (GLOX) from Pycnoporus cinnabarinus, have also been reported to act on furfural derivatives, but their activity is restricted to the alcohol or the aldehyde groups, respectively (15). A recent study has demonstrated the ability of aryl-alcohol oxidase (AAO) from Pleurotus eryngii to catalyze the separate oxidation of HMF, DFF, and FFCA, although the complete oxidation of HMF by AAO stops at the FFCA level, due to inhibition of the last oxidation step by the peroxide generated in the first two reactions (16). More recently, a new copper radical oxidase from Colletotrichum graminicola has been described as an AAO-type enzyme and reported to oxidize HMF into DFF and 5-hydroxymethylfurancarboxylic acid (HMFCA) into FFCA without detected activity on the aldehyde groups of these compounds (17). Due to the above-named characteristics, efficient production of FDCA by the latter oxidases requires the combination of several enzymes, such as the AAO/chloroperoxidase (18), AAO/peroxygenase (15, 19), GAO/peroxygenase (15, 20), GLOX/AAO (21), or AAO/catalase (16) couples already reported. Therefore, *Metsp*HMFO is the only enzyme described to date able to perform by itself (i.e., without the concourse of a second enzyme) the three oxidation steps for FDCA production from HMF. In recent years, several strategies have been followed to increase the performance of *Metsp*HMFO for FDCA bioproduction, such as its coexpression with the HmfH enzyme, which oxidizes HMF by the HMFCA route (3), in whole-cell systems (22), directed mutagenesis of *Metsp*HMFO (23), or its combination with lipase in an enzymatic cascade (24).

Taking into account the good performance and promising use of HMFO for FDCA production, in the present study, we focused first on the search for new HMFOs in databases. Then, as a result of Escherichia coli expression trials with four HMFO genes, two new enzymes, from Pseudomonas nitroreducens and an unidentified *Pseudomonas* strain (25), were purified and characterized, and their oxidative activity on HMF was analyzed in short- and long-term reactions.

## RESULTS

### Identification of new HMF-oxidizing enzymes.

**FIG 2:**
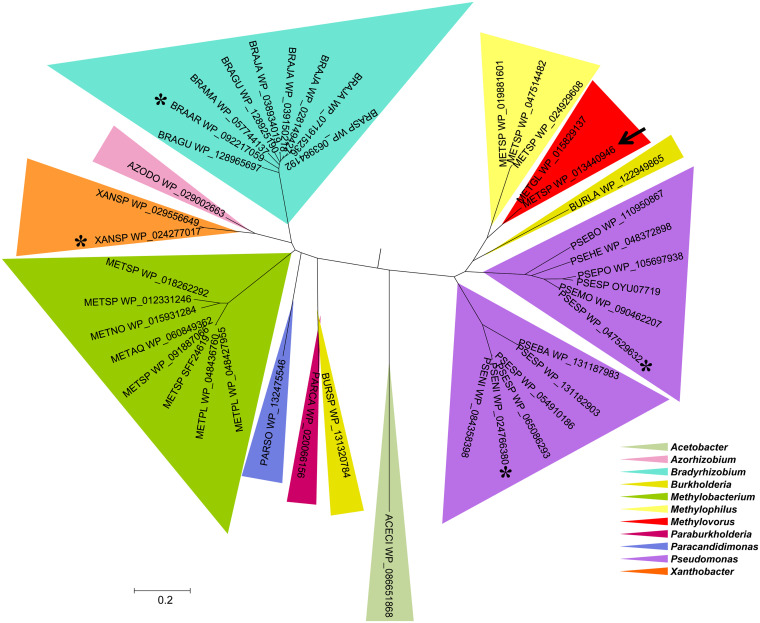
Maximum-likelihood phylogenetic tree of 41 HMFO-like sequences in 11 bacterial genera, from searching GenBank. The sequence used as the BLAST query is indicated with an arrow, and the four sequences optimized for E. coli expression are indicated with asterisks.

BLAST of the amino acid sequence of *Metsp*HMFO (UniProt E4QP00) against the nonredundant protein sequences in GenBank revealed 41 entries with identities of >45%, all of them from the phylum *Proteobacteria* (Fig. 2). The largest numbers of HMFOs were found in the *Pseudomonas* (12 sequences), *Bradyrhizobium* (9 sequences), and *Methylobacterium* (8 sequences) genera, and those with the highest homology with *Metsp*HMFO were HMFOs from *Methylophylus*, *Pseudomonas*, and *Burkholderia* species, with more than 60% identity with the query (Table S1 in the supplemental material).

**FIG 3:**
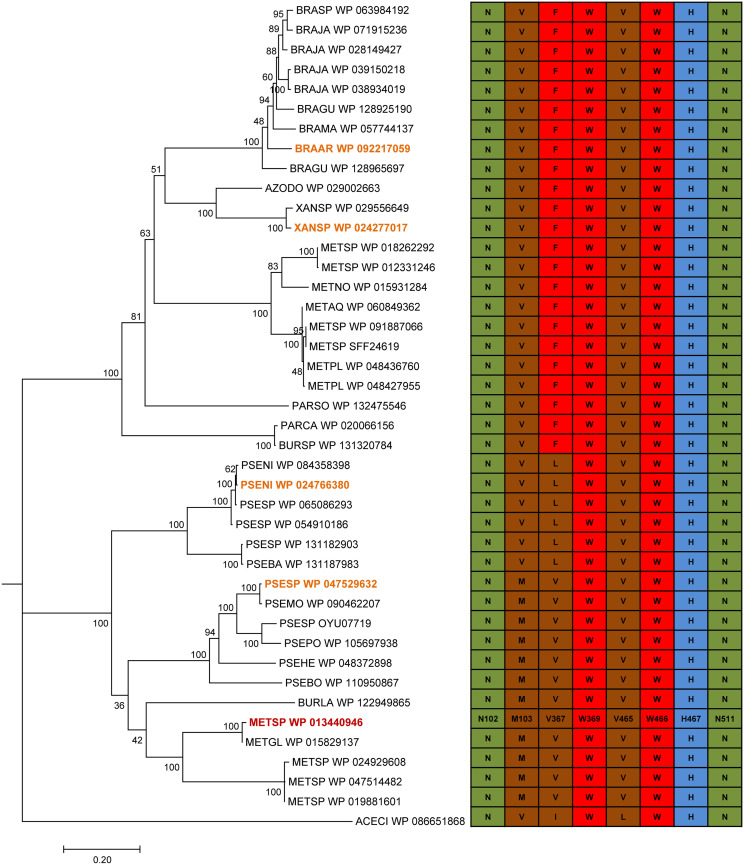
Conservation of residues around the HMFO active site after alignment of sequences included in the phylogenetic tree. Nonpolar residues are colored in brown, aromatic residues are colored in red, basic residues are colored in blue, and acidic residues are colored in green. The four new HMFO sequences optimized for gene expression in E. coli and that of *Metsp*HMFO are labeled in orange and red, respectively.

All of the sequences described above conserve the ADP-binding domain and the consensus sequences PS00623 and PS00624, typical of the GMC oxidoreductase superfamily (Fig. S1) (1). A more detailed analysis of conserved active-site amino acids (Fig. 3) reveals that all of them include the same histidine and asparagine residues, *Metsp*HMFO His467 and Asn511, at the catalytic positions described for the latter enzyme (12). Asn102, Trp369, and Trp466 are also fully conserved, while substitutions are often observed at Met103, involved in the correct orientation of the substrate with respect to the active site, and at Val367, described as a site for improving the activity on carboxyl-containing substrates (12). From this repertoire of enzymes, four HMFO sequences from the two main HMFO clusters (Fig. 2)—one from P. nitroreducens (*Pseni*HMFO), one from *Pseudomonas* sp. strain 11/12A (*Psesp*HMFO), one from *Xanthobacter* sp. strain 126 (*Xansp*HMFO), and one from Bradyrhizobium arachidis (*Braar*HMFO)—were selected and optimized for E. coli expression to produce them as recombinant proteins, together with *Metsp*HMFO.

### Expression and purification of two new HMFOs.

The four optimized genes (Table S2) were cloned into the pET23b and/or the His-tagged pET28a plasmid and transformed into E. coli C41(DE3). *Xansp*HMFO and *Braar*HMFO could not be expressed using either the pET23b or the pET28a vector, while *Pseni*HMFO and *Psesp*HMFO were purified and characterized after pET28a and pET23b expression, respectively. To evaluate whether the His tag affects the properties of HMFOs, the optimized gene encoding *Metsp*HMFO (Table S2) was also cloned into both plasmids, and the tagged/untagged forms were characterized in parallel. The four proteins were expressed as soluble, active enzymes by slow induction at 16°C during 72 to 96 h.

Untagged *Metsp*HMFO and *Psesp*HMFO were purified to electrophoretic homogeneity (Fig. S2a and c, respectively) by two anion exchange chromatographic steps (see conditions in Table S3), yielding 34 and 10 mg of protein per liter of culture, respectively (Table 1). For the His-tagged proteins, anionic chromatography after the affinity chromatographic step provided pure enzyme (Fig. S2b and d), with yields of 17 and 47 mg of protein per liter of culture of *Metsp*HMFO^His^ and *Pseni*HMFO^His^, respectively (Table 1). The specific activities of the purified enzymes were 3.3 to 3.6 U · mg^−1^ for *Metsp*HMFO (similar for the untagged and the His-tagged forms), 2.0 U · mg^−1^ for *Psesp*HMFO, and 0.2 U · mg^−1^ for *Pseni*HMFO^His^. Vanillyl alcohol was used for the activity estimations described above, since it is oxidized with the highest catalytic efficiency, as described below.

**TABLE 1:** Summary of purification processes of recombinant HMFOs^*a*^

HMFO	Purification step	Total protein (mg)	Total activity (U)^*b*^	Sp act (U/mg)	Degree of purification (fold)	Yield (%)
*Metsp*HMFO	Crude extract	1,660	284	0.17	1.0	100
	ResourceQ	71	187	2.62	15.4	66
	MonoQ	34	120	3.57	21.0	42
*Metsp*HMFO^His^^*c*^	Crude extract	1,910	100	0.05	1.0	100
	HiTrap	31	81	2.65	50.4	81
	MonoQ	17	56	3.30	62.9	56
*Psesp*HMFO	Crude extract	1,720	138	0.08	1.0	100
	ResourceQ	50	71	1.42	17.8	52
	MonoQ	10	20	2.02	25.2	15
*Pseni*HMFO^His^^*c*^	Crude extract	1,650	12	0.01	1.0	100
	HiTrap	103	10	0.09	12.6	79
	MonoQ	47	8	0.16	22.2	63

a Data are for protein from 1 liter of E. coli culture.

b Measured with vanillyl alcohol in 50 mM Tris-HCl, pH 7.0, at 25°C.

c His-tagged protein.

The molecular weights (MW) of the different HMFOs, measured by matrix-assisted laser desorption and ionization-time of flight (MALDI-TOF) mass spectrometry, were ∼57 kDa (Table 2), in good agreement with the theoretical masses calculated from their amino acid sequences (Table S1). This indicates that HMFOs are not purified as covalently bound dimeric enzymes. Also, the experimental isoelectric point (pI) values of 6.0 to 6.5 were similar to the theoretical ones (as shown in the tables mentioned above).

**TABLE 2:** Spectroscopic and physicochemical properties of recombinant HMFOs^*a*^

HMFO	Value for indicated property						
	Spectroscopic^*b*^				Physicochemical^*c*^		
	λ_band II_ (nm)	λ_band I_ (nm)	ε_band I_ (M^−1^ cm^−1^)	*A*_278_/*A*_band I_	MW (Da)	pI	Mean *T_m_* ± SD (°C)
*Metsp*HMFO	387	457	11,271	10.7	57,547.0	6.5	47.93 ± 0.02
*Metsp*HMFO^His^	387	457	11,667	10.9	57,854.3	6.6	47.77 ± 0.06
*Psesp*HMFO	387	457	11,432	10.4	56,945.2	6.1	40.05 ± 0.18
*Pseni*HMFO^His^	382	456	12,060	10.1	58,863.5	6.5	43.80 ± 0.21

a Measured in 50 mM Tris-HCl, pH 7.0, at 25°C.

b λ_band_, wavelength of the corresponding flavin band; ε_band_, extinction coefficient at the corresponding flavin band. See Fig. S3b.

c MW, pI, and *T_m_* values were estimated by MALDI-TOF, isoelectric focusing, and ThermoFAD assay, respectively.

### HMFO spectral properties.

The secondary structures of the enzymes were evaluated by circular dichroism (CD) (Fig. S3a). The far-UV spectra showed minimums at ∼222 nm, typical of α-helix structures, being more intense in the *Metsp*HMFO and *Psesp*HMFO spectra. The contents in α-helixes (27 to 47%), β-sheets (11 to 20%), and turns (20 to 24%) were obtained by deconvolution of the CD spectra (Table S4). As shown for *Metsp*HMFO^His^, the molar ellipticity per residue (MER) intensity revealed an α-helix decrease (from 37% to 27%) and an increase of unordered structure (from 26% to 37%) in the His-tagged enzymes.

The enzymes showed UV-visible spectra characteristic of FAD-containing proteins (Table 2 and Fig. S3b). Two peaks at 382 to 387 nm (band II) and 456 to 457 nm (band I) with a shoulder at 483 nm and *A*_279_/*A*_457_ ratios of ∼10 were observed, indicating the proper incorporation of the cofactor. The calculated extinction coefficients at the band I maximums (ε_band I_) of *Metsp*HMFO, *Metsp*HMFO^His^, *Psesp*HMFO, and *Pseni*HMFO^His^ were 11,271, 11,667, 11,432, and 12,060 M^−1^ cm^−1^, respectively. These values were used to estimate the corresponding enzyme concentrations.

### Temperature and pH stability.

**FIG 4:**
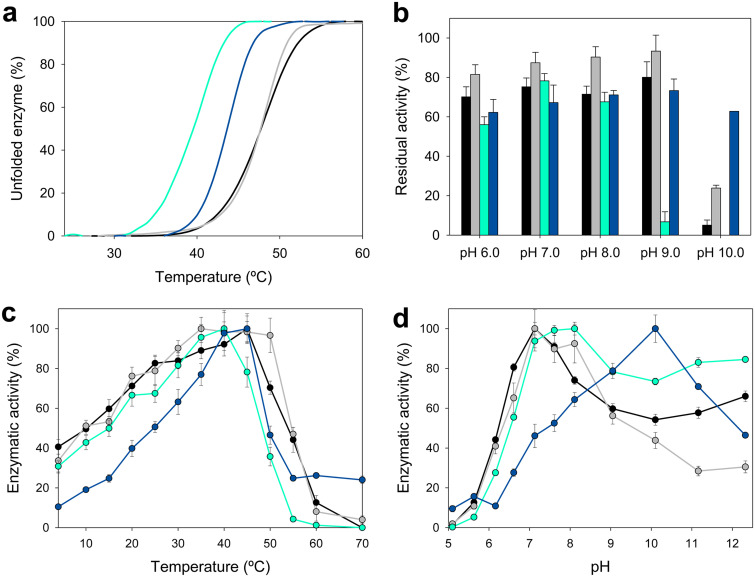
Thermal stability measured as melting temperature (a), pH stability after 72-h incubation at 25°C in B&R buffer (pH 6 to 9) (b), and optimal temperature (c) and pH (d) (referred to the optimal value) for *Metsp*HMFO (black), *Metsp*HMFO^His^ (gray), *Psesp*HMFO (cyan), and *Pseni*HMFO^His^ (blue). All activities (b to d) were measured with vanillyl alcohol as the substrate. Mean values and standard deviations are shown.

As an estimation of thermostability, the melting temperature (*T_m_*) for each enzyme was calculated from the changes of FAD fluorescence using the ThermoFAD assay (26). The denaturation curves indicated a *T_m_* of 48°C for the enzyme from *Methylovorus* (both *Metsp*HMFO and *Metsp*HMFO^His^), while *Psesp*HMFO and *Pseni*HMFO appeared to be 8°C and 4°C less stable, respectively (Fig. 4a and Table 2).

To determine the range of pHs in which the enzymes are active, we measured the residual activity on vanillyl alcohol after 72 h of incubation under different pH conditions. The enzyme from *Methylovorus* (both *Metsp*HMFO and *Metsp*HMFO^His^) was quite stable at pH 6 to 9, conserving 70 to 85% activity (Fig. 4b). At pH 10, the activities of *Metsp*HMFO and *Metsp*HMFO^His^ decreased to only 5% and 24% activity, respectively, while *Pseni*HMFO^His^ kept ∼65 to 70% of its initial activity between pH 6 and 10. The range in which *Psesp*HMFO was stable was slightly narrower, pH 6 to 8, with only ∼10% activity and total activity loss at pH 9 and pH 10, respectively. Outside these pH ranges, the activity of all the enzymes decreased abruptly in the first 24 h (data not shown).

### Optimal temperature and pH.

The pH and temperature optima were also calculated with vanillyl alcohol as the substrate. *Psesp*HMFO and *Pseni*HMFO^His^ attained their maximal activities at 35 to 40°C and 40 to 45°C, respectively, and decreased to 50% below 15°C and 25°C, respectively, and above 50°C for both (Fig. 4c). Similar profiles were observed for *Metsp*HMFO, which attained its maximal activity at 45°C and kept more than 80% activity between 25 and 45°C (20 to 50°C for *Metsp*HMFO^His^).

With regard to pH, none of the enzymes were active below pH 5 (Fig. 4d). The optimum for *Metsp*HMFO (both for the untagged and His-tagged enzyme) was at pH 7.0, with the enzyme retaining ∼70% of its activity at pH 6.5 to 8.0. Similarly, *Psesp*HMFO showed its maximal activity at pH 7.5 to 8.0 and still showed 80% of its activity at pH 7 to 12. Although at basic pHs, the initial rates for *Metsp*HMFO and *Psesp*HMFO were high, the velocities decayed abruptly after a few seconds due to enzyme inactivation. However, *Pseni*HMFO^His^ exhibited a quite different pH behavior, since it was more active at basic pH, with the maximal value at pH 10.0. Below pH 9.0 and above pH 11.0, its activity decreased to less than 80%.

### Effect of H_2_O_2_ on HMFOs’ stability.

**FIG 5:**
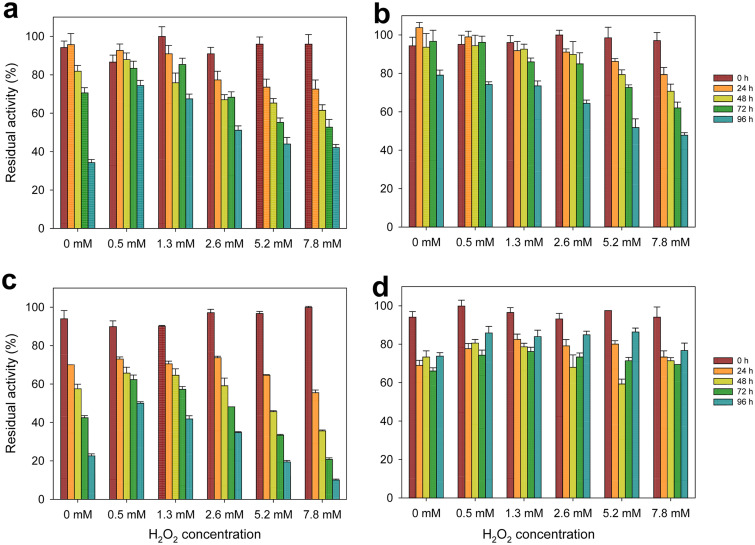
Effect of H_2_O_2_ on the stability of *Metsp*HMFO (a), *Metsp*HMFO^His^ (b), *Psesp*HMFO (c), and *Pseni*HMFO^His^ (d). Residual activities were measured with vanillyl alcohol after different incubation times with increasing amounts of H_2_O_2_ in 50 mM Tris-HCl, pH 7.0, at 25°C. Mean values and standard deviations are shown.

Since during HMFO activity, hydrogen peroxide is produced from O_2_ reduction, we evaluated the effect that different amounts of H_2_O_2_ (0.5 to 7.8 mM) had on the enzymes’ stability. The results for *Metsp*HMFO and *Metsp*HMFO^His^ suggest that the His tag has a positive effect on enzyme stability, even in the absence of H_2_O_2_ (Fig. 5a and b). Also, *Pseni*HMFO^His^ was more stable to peroxide exposure than *Metsp*HMFO, keeping around 70% of its activity after 96 h in all the concentrations tested (Fig. 5d). In contrast, *Psesp*HMFO was the least stable enzyme (Fig. 5c). In fact, low peroxide amounts (0.5 to 2.6 mM) had a slight stabilizing effect on *Psesp*HMFO, while at the highest concentration, its activity decreased to only 10%.

### Kinetic parameters for oxidation of HMF and its DFF and FFCA derivatives.

The kinetic parameters for oxidation of vanillyl alcohol and furfuryl substrates HMF, DFF, and FFCA were determined for the four enzyme preparations (Table 3). The untagged and His-tagged *Metsp*HMFO showed similar *k*_cat_ and *K_m_* values for vanillyl alcohol (measured by direct spectrophotometric estimation of the aldehyde product). *Psesp*HMFO oxidized vanillyl alcohol with a catalytic efficiency (*k*_cat_/*K_m_*) similar to that of the enzyme from *Methylovorus* sp., while *Pseni*HMFO^His^ was less efficient in oxidizing this substrate (with ∼28-fold-lower values than the other enzymes).

**TABLE 3:**
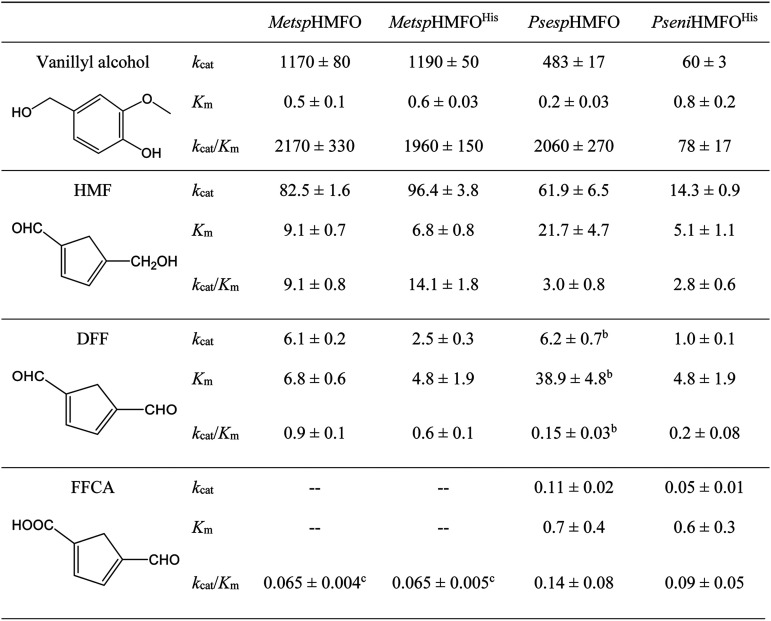
Steady-state kinetic constants for the oxidation of vanillyl alcohol, HMF, DFF, and FDCA by HMFOs^*a*^

a Measured at 25°C in 50 mM Tris-HCl, pH 7.0, except for vanillyl alcohol oxidation by *Psesp*HMFO and *Pseni*HMFO^His^, which were measured at optimal pHs of 8 and 10, respectively. Units of measure are as follows: *k*_cat_, min^−1^; *K_m_*, mM; *k*_cat_/*K_m_*, mM^−1^ min^−1^.

b Data fitted to Hill equation (*n* = 2.4 ± 0.5).

c Due to substrate inhibition, *Metsp*HMFO and *Metsp*HMFO^His^ were not saturated and only a *k*_obs_/[FFCA] value was obtained.

Oxidation of HMF and DFF was followed by measuring H_2_O_2_ release, using a coupled assay with horseradish peroxidase (HRP) at pH 7.0 (which is close to the HRP optimum). HMF was the best furfuryl substrate for all the enzymes. *Metsp*HMFO and *Psesp*HMFO showed similar *k*_cat_ values, with a significant increase in the *K_m_* for *Psesp*HMFO, while for *Pseni*HMFO^His^, the *K_m_* was lower. The efficiencies for HMF oxidation by *Psesp*HMFO and *Pseni*HMFO were ∼4-fold lower than for *Metsp*HMFO. Concerning DFF oxidation, it was in all cases 10- to 14-fold slower than that of HMF, showing that the *Psesp*HMFO enzyme had a positive cooperative effect (Hill coefficient, *n_H_* = 2.4) and, again, a *K_m_* value significantly higher than for the rest of the enzymes.

Due to the very slow FFCA oxidation, the kinetic parameters for this compound could not be calculated in a continuous assay. Therefore, the production of FDCA was followed by high-performance liquid chromatography (HPLC) during 48 h of incubation with increasing concentrations of FFCA at 25°C and pH 7.0. The enzyme from *Methylovorus* showed strong substrate inhibition at FFCA concentrations higher than 3 mM. This made it impossible to assess the kinetic parameters, as no saturation by FFCA was attained and only *k*_obs_ (observed rate constant)/concentration values were estimated for comparison of efficiencies. The FFCA inhibitory effect was much milder for the enzymes from *Pseudomonas*, and saturation was attained due to the lower *K_m_* values. These results showed that the FFCA oxidation efficiencies of *Pseni*HMFO^His^ and *Psesp*HMFO are 1.4- and 2.2-fold higher, respectively, than those of *Metsp*HMFO. However, the differences in catalytic efficiencies for FFCA oxidation with regard to those for HMF and DFF were significantly lower in *Pseni*HMFO^His^ and *Psesp*HMFO than in *Metsp*HMFO. This suggests a more efficient overall oxidation of HMF to produce FDCA by the *Pseudomonas* enzymes.

### HMF conversion: effects of pH and temperature.

**FIG 6:**
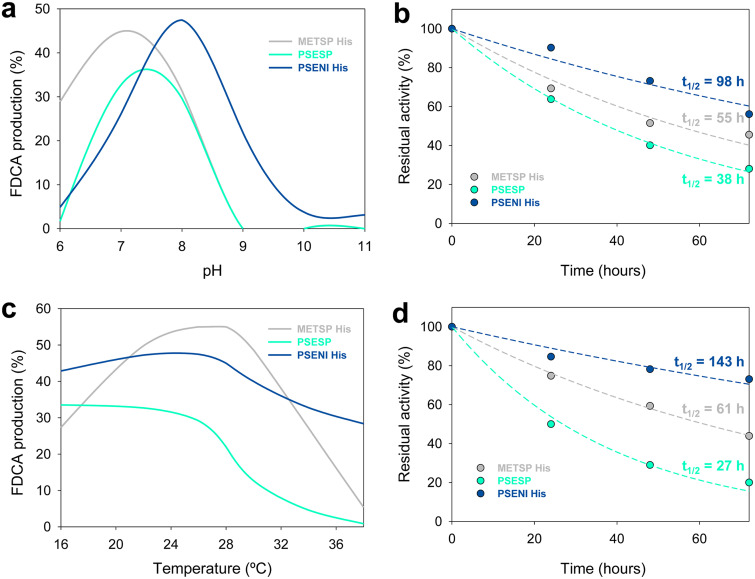
FDCA production after 72-h reaction of 1.5 mM HMF with 2.5 μM *Metsp*HMFO^His^ (gray), *Psesp*HMFO (cyan), and *Pseni*HMFO^His^ (blue) under different pH (a) and temperature (c) conditions, and residual activities along the reactions at the optimal pH (b) and 28°C temperature (d).

Taking into account the similar kinetic parameters of *Metsp*HMFO and *Metsp*HMFO^His^ and the higher stability under exposure to H_2_O_2_ of the latter form, the following experiments with this enzyme were performed with the His-tagged form. As for *Metsp*HMFO^His^, the enzymes from *Pseudomonas* were able to oxidize HMF to FDCA, catalyzing the three reaction steps. Taking into account their pH and temperature profiles (Fig. 4c and d), the conditions for HMF conversion by each enzyme were investigated in 72-h reactions (Fig. 6). Maximal FDCA production was achieved at pH 7.0, pH 7.5, and pH 8.0 for *Metsp*HMFO^His^, *Psesp*HMFO, and *Pseni*HMFO^His^, respectively (Fig. 6a). Under optimal pH conditions, *Metsp*HMFO^His^ and *Psesp*HMFO showed half-lives (*t*_1/2_) of 55 and 38 h, respectively, while *Pseni*HMFO^His^ was more stable, with a remarkable *t*_1/2_ of ∼100 h (Fig. 6b). Outside their optimal pH conditions, *Metsp*HMFO^His^ and *Psesp*HMFO had reduced half-lives, while *Pseni*HMFO^His^ was quite stable in the range of pH 6 to 8 (Fig. S4, left).

Concerning temperature, *Pseni*HMFO^His^ and *Psesp*HMFO kept maximal FDCA production (around 45% and 30%, respectively) in the range of 16 to 28°C, while *Metsp*HMFO^His^ showed maximal production in the 24 to 28°C range (Fig. 6c) (all reactions at optimal pH). At 28°C, the *t*_1/2_ values for the three HMFOs were 61, 27, and 143 h for *Metsp*HMFO^His^, *Psesp*HMFO, and *Pseni*HMFO^His^, respectively (Fig. 6d). Above 28°C, *Metsp*HMFO^His^ and *Psesp*HMFO had decreased FDCA yields (Fig. 6c) due to enzyme inactivation, as shown by the *t*_1/2_ decreases (Fig. S4, right). *Pseni*HMFO^His^ was the most thermostable enzyme for FDCA production (Fig. S4f), with *t*_1/2_ values of more than 100 h in the range of 16 to 28°C and around 90 h at 30 and 34°C. Even at 38°C, where the other two enzymes showed *t*_1/2_ values of ≤4 h, *Pseni*HMFO^His^ had a *t*_1/2_ of 15 h.

### FDCA production: kinetics and long-term conversions.

**FIG 7:**
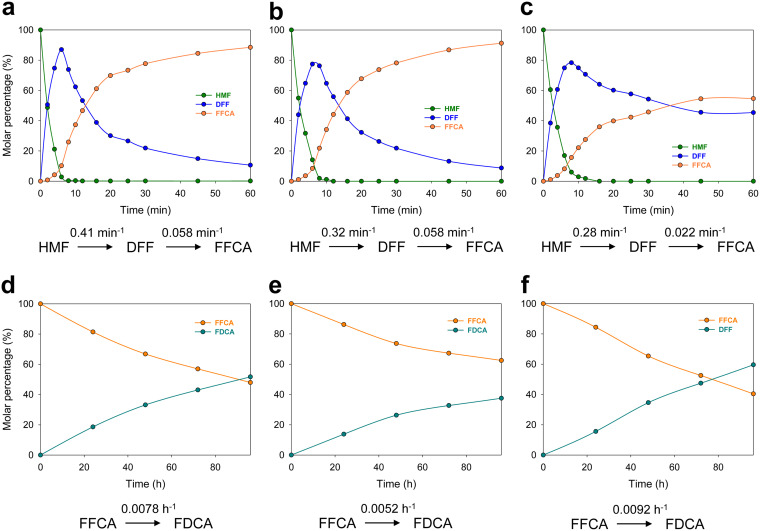
Time course of reaction of 1.5 mM HMF (a to c) or FFCA (d to f) with 2.5 μM *Metsp*HMFO^His^ (a, d), *Psesp*HMFO (b, e), or *Pseni*HMFO^His^ (c, f) in 50 mM Tris-HCl at the optimal pH for each enzyme and 25°C. Oxidation rates, estimated by fitting to equations $\left[\text{HMF}\right]={\left[\text{HMF}\right]}_{0}e^{-k_{1}t}$, $\left[\text{DFF}\right]={\left[\text{HMF}\right]}_{0}\left[k_{1}/\left(k_{2}-k_{1}\right)\right]\left(e^{-k1t}-e^{-k_{2}t}\right)$, and $\left[\text{FFCA}\right]={\left[\text{FFCA}\right]}_{0}e^{-k_{1}t}$, are indicated.

Taking into account the results described above, oxidation of HMF by the different HMFOs was followed first during 1 h at 25°C and the optimal pH of each enzyme (Fig. 7). For all of them, the oxidation of HMF was faster than that of DFF [*k*_(HMF→DFF)_/*k*_(DFF→FFCA)_ was ∼7-, 6-, and 13-fold faster for *Metsp*HMFO^His^, *Psesp*HMFO, and *Pseni*HMFO^His^, respectively], with *Pseni*HMFO^His^ being the least efficient in these conversions, as expected from the HMF and DFF steady-state parameters (Table 3). For FFCA oxidation, reactions were followed during 4 days under the same conditions. As expected, this reaction was seen to be the limiting step for FDCA production, with *k*_(FFCA→FDCA)_ values of 0.0078, 0.0052, and 0.0092 h^−1^ for *Metsp*HMFO^His^, *Psesp*HMFO, and *Pseni*HMFO^His^, respectively, indicating a more efficient FFCA oxidation by *Pseni*HMFO^His^, which would therefore be the best enzyme for application.

**FIG 8:**
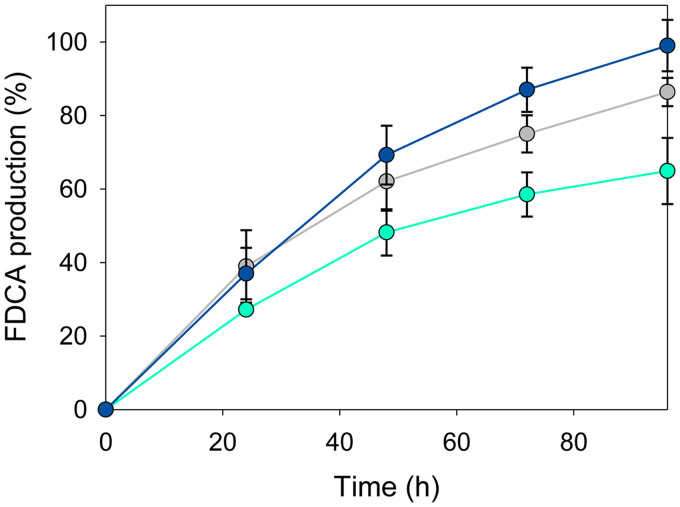
FDCA production from HMF (1.5 mM) by the *Metsp*HMFO (gray), *Psesp*HMFO (cyan), and *Pseni*HMFO (blue) enzymes (5 μM) in 50 mM Tris-HCl at 28°C and the optimal pH for each of them. Mean values and standard deviations are shown.

Finally, long-term conversion of HMF was followed during 4 days (Fig. 8) at 28°C and the optimal pH for each enzyme. With a substrate/enzyme molar ratio of 300, the highest FDCA production was achieved by *Pseni*HMFO^His^, with nearly complete conversion into FDCA after 96 h of reaction, while for *Metsp*HMFO^His^ and *Psesp*HMFO, the yields were 89% and 65%, respectively (Table 4). Interestingly, under these conditions, *Pseni*HMFO^His^ showed a significantly longer half-life (150 h) than the other enzymes (∼40 to 60 h) and a higher total turnover number (TTN) for FDCA production.

**TABLE 4:** Catalytic performance parameters for the production of FDCA from HMF by HMFOs in Tris-HCl^*a*^

HMFO	Optimal pH	Half-life (h)	TTN^*b*^	% FDCA production (mean ± SD)
*Metsp*HMFO^His^	7.0	61	802	89 ± 4
*Psesp*HMFO	7.5	40	584	65 ± 9
*Pseni*HMFO^His^	8.0	150	891	99 + 7

a Production of FDCA from HMF at 1.5 mM by HMFOs at 5 μM in 50 mM Tris-HCl at 28°C after 96 h of reaction.

b TTN, total turnover number.

## DISCUSSION

### HMFO screening, heterologous expression, and purification.

The HMFO from *Methylovorus* sp. (*Metsp*HMFO) is the only enzyme described to date that is directly related to HMF oxidation. This connection was established due to its homology with the related HmfH protein that forms part of a gene cluster involved in HMF metabolism in C. basilensis (3, 6). In the present study, new HMFOs from P. nitroreducens and a *Pseudomonas* sp. strain were described, following their production as recombinant proteins. These two HMFOs, together with others from a *Xanthobacter* sp. strain and B. arachidis that could not be produced in E. coli, were selected from a total of 41 HMFO-like sequences analyzed after a database search for homology with *Metsp*HMFO. Although an earlier attempt to express the gene responsible for HMF oxidation by C. basilensis and a homologous gene from Paraburkholderia phytofirmans was made, only *Metsp*HMFO had been successfully produced as a functional protein (6) to date. Here, the two sequences from *Pseudomonas* strains, with 63 to 64% identity with the *Metsp*HMFO sequence, were heterologously expressed in E. coli as active, soluble enzymes, together with *Metsp*HMFO. The three recombinant HMFOs appeared as monomeric proteins, in agreement with a previous report on the latter enzyme (12), and were purified with good yields, increasing to three the number of members of the HMFO family currently available.

### New information on *Metsp*HMFO.

Comparison of the results obtained here for the *Methylovorus* enzyme purified without and with a His tag (*Metsp*HMFO and *Metsp*HMFO^His^) indicated that the tag does not perturb the incorporation of the cofactor, as shown by the UV-visible spectra. Moreover, deconvolution of the CD spectra indicated an increase in the unordered structure, consistent with 90% of the His tag being disordered (27), and a slight decrease in the α-helix content. Neither activity nor the main physicochemical parameters were affected by the presence of the His tag, but a positive effect on stability under exposure to H_2_O_2_ was found for the tagged *Metsp*HMFO enzyme. This would represent an advantage for enzymes generating H_2_O_2_, which, as reported for other oxidases of the same superfamily, can inhibit the enzyme reactions or the enzymes themselves (28, 29).

The kinetic parameters for vanillyl alcohol oxidation by *Metsp*HMFO were similar to those previously reported for the same enzyme (14). However, the reported *k*_cat_ values for HMF and DFF were significantly higher (594 min^−1^ and 96 min^−1^, respectively) than those obtained in the present study. Considering the steady-state kinetic parameters obtained here, the estimated times for HMF and DFF consumption would be 7 and 98 min, respectively, which is consistent with the results obtained in 1-h reactions (1.5 mM HMF and 2.5 μM HMFO). In contrast, Dijkman et al. (14) still detected DFF after 1 h of reaction between 2 mM HMF or DFF and 1 μM HMFO, while according to their *k*_cat_ value, all DFF should be consumed in ∼20 min. In any case, the *k*_cat_ and *K_m_* values for oxidation of these furfural derivatives by the *Metsp*HMFO produced here are of the same order as those previously reported for other oxidases acting on these substrates, such as AAO (19) and GLOX (21). Regarding FFCA, the catalytic efficiency, estimated as *k*_obs_/[FFCA], was of the same order as that previously reported for *Metsp*HMFO (12), although the strong inhibition by FFCA (above 3 mM) has not been described before.

### Properties of two new HMFOs.

The new enzymes from *Pseudomonas* strains were able to directly produce FDCA from HMF, with slight differences in their optimal reaction conditions, such as the higher activity and stability of *Pseni*HMFO under slightly alkaline conditions. Although the new HMFOs do not show more activity on HMF and DFF than *Metsp*HMFO, it is important to mention that they show higher efficiencies for FFCA oxidation, the rate-limiting step in FDCA production by HMFO (14) and other oxidases (16).

Strikingly, in terms of efficiency, *Psesp*HMFO showed values for FFCA oxidation similar to those observed for DFF oxidation. The cooperative effect for DFF oxidation by this enzyme can be attributed to slow conformational changes that accompany substrate binding or product release. Similar cooperative effects have been described for other monomeric enzymes with single ligand-binding sites (30, 31).

In the case of *Pseni*HMFO, the efficiency for FFCA oxidation was only half of that of DFF oxidation. The results described above contrast with the much lower efficiency observed for *Metsp*HMFO (FFCA oxidation 10-fold lower than DFF oxidation). The comparatively higher speed in rate-limiting FFCA oxidation by *Pseni*HMFO results in higher final yields of FDCA in HMF bioconversions under optimized conditions. Moreover, its higher tolerance of the H_2_O_2_ produced during the three oxidation steps results in a higher enzymatic stability during turnover, increasing its half-life. This stability provides an important advantage, since oxidases are often inhibited by large amounts of H_2_O_2_. Thus, our data reveal *Pseni*HMFO as a robust and suitable biocatalyst for prolonged incubations, due to its longer half-life in wider ranges of pHs, temperatures, and H_2_O_2_ concentrations.

### Analysis of HMFO molecular models.

Molecular modeling of *Psesp*HMFO and *Pseni*HMFO was undertaken at the Swiss-Model server (32), using the *Metsp*HMFO crystal structure (PDB code 4UDP) (12) as the template. The predicted secondary structures were similar, as expected from their far-UV CD spectra.

**FIG 9:**
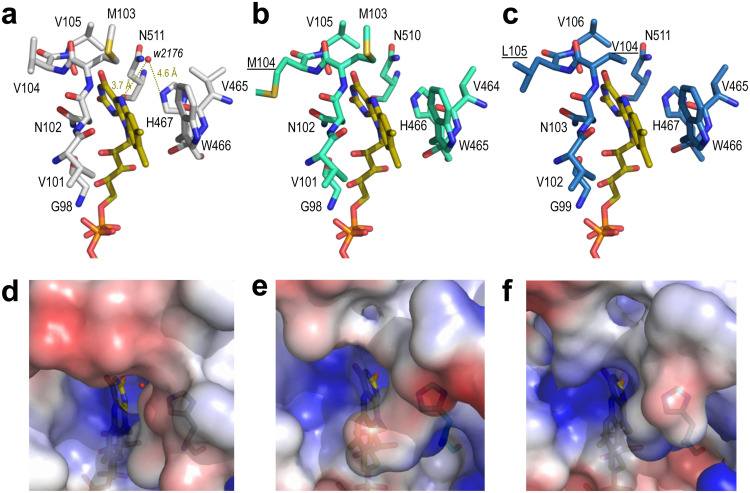
Details of the active sites of *Metsp*HMFO (a, d), *Psesp*HMFO (b, e), and *Pseni*HMFO (c, f). (a to c) Residues at less than 6 Å from the reactive N5 atom of the FAD cofactor (CPK-colored sticks) are indicated, with those differing from *Metsp*HMFO underlined. Distances from His467 and flavin ring to water2176 (red sphere) are shown in panel a. (d to f) Semitransparent solvent-accessible surfaces colored by electrostatic potential (red, negative; blue, positive) around the flavin access channel. Water2176 inside the channel is shown in panel d. From the crystal structure with PDB code 4UDP (12) (a and d) and molecular homology models (32) (b, c, e, and f).

However, inspection of their active sites revealed subtle differences in the environment (less than 6 Å) of the reactive N5 of FAD. Interestingly, in the *Metsp*HMFO crystal structure, a water molecule is located at 3.7 Å from the above-named N atom and 4.6 Å from the catalytic His467 (Fig. 9a), at a position that is most probably occupied by the substrates during catalysis. Concerning neighbor residues, the pair Met103-Val104 in *Metsp*HMFO (Fig. 9a) is replaced by Met103-Met104 in *Psesp*HMFO (Fig. 9b) and by Val104-Leu105 in *Pseni*HMFO (Fig. 9c). These two positions follow an active-site asparagine that is totally conserved in the GMC superfamily (1) as part of the conserved PS00623 sequence. Directed mutagenesis of *Metsp*HMFO revealed how an M103A substitution (a changed of Met to Ala at position 103) significantly modified the enzyme turnover (for vanillyl alcohol) due to substrate pocket modification (12). A free space, potentially affecting the position of HMF and its partially oxidized derivatives (DFF and FFCA) with respect to the flavin cofactor, could also exist in *Pseni*HMFO with a valine at this position.

The above-described differences do not include the presence of residues that could promote carbonyl activation in FFCA (33), as found in classical aldehyde-oxidizing oxidoreductases. However, changes in the flavin environment of *Psesp*HMFO and *Pseni*HMFO could modify the interaction of the flavin with the protein. Such interactions can modulate the redox potential of the oxidized isoalloxazine ring, promoting the enzyme’s activity on less reactive substrates (34).

Pointing to the active-site cavity, Val367 is found in *Metsp*HMFO. This residue is conserved in *Psesp*HMFO, while Leu367 replaces the valine in *Pseni*HMFO (Fig. 3). Interestingly, it has been reported that mutating this valine to arginine in *Metsp*HMFO results in higher activity on FFCA (12), as found for *Pseni*HMFO. However, the effect in the *Metsp*HMFO variant is due to the introduction of a basic (arginine) residue, contributing to adequate positioning of the above-mentioned acidic substrate, while the natural change in *Pseni*HMFO would only slightly reduce the pocket size.

On the other hand, the electrostatic surface potentials of the three HMFOs showed differences in charge distribution around the entrance of the flavin-access channel (Fig. 9d to f), which could also modulate the access of substrates. Namely, the electronegative region partially covering the channel entrance in *Metsp*HMFO could impede the access of acidic FFCA to the active site. However, the less electronegative entrances in *Psesp*HMFO and especially in *Pseni*HMFO would facilitate the entrance of this substrate, resulting in the observed higher activity of the new enzymes in catalyzing the rate-limiting step in HMF conversion to FDCA. The solvent access surfaces also showed that, although the O_2_ substrate would more easily diffuse to attain the flavin ring of the buried cofactor in HMFOs, the entrance of the furfuryl-reducing substrates through the narrow access channel would most probably involve some side chain rearrangement at the residues forming the channel, as described in a related AAO (35, 36).

### Concluding remarks.

Two enzymes from two *Pseudomonas* species have been added to the repertoire of HMFOs available, where only one member was purified and characterized to date. The enzyme from P. nitroreducens is a promising candidate for HMF oxidation due to its higher catalytic efficiency for FFCA oxidation, the bottleneck for FDCA production, compared to that of the previously described *Metsp*HMFO. More importantly, in the context of industrial production of FDCA, additional advantages of the P. nitroreducens enzyme are its stability under exposure to H_2_O_2_ and its robustness for long-term incubations, as shown by its high *t*_1/2_ values under wider ranges of pH and temperature conditions. This feature would facilitate the enzyme’s reuse and applicability in continuous operation for the production of FDCA as a building block for the production of bioplastics.

## MATERIALS AND METHODS

### Chemicals.

HMF was kindly provided by AVA Biochem. DFF, FDCA, vanillyl alcohol, and vanillin were purchased from Sigma-Aldrich (St. Louis, MO, USA). FFCA was purchased from TCI America (Portland, OR, USA). AmplexRed and horseradish peroxidase were obtained from Invitrogen (Waltham, MA, USA).

### Sequence search, phylogenetic analysis, and structure modeling.

The screening of HMFO-like sequences was performed by BLAST of the amino acid sequence of *Metsp*HMFO against the nonredundant protein sequences in GenBank. Multiple alignments of the enzymes found with Clustal Omega (37) were used to identify the conserved motifs (ADP binding domain and Prosite PS00623 and PS00624 sequences) in GMC proteins. A maximum-likelihood phylogenetic tree was constructed by MEGA X with 100-iteration bootstrapping (38) using the Whelan and Goldman (39) model of evolution using gamma-distributed rate variation with empirical amino acid frequencies and invariant sites (WAG+F+I+G). Finally, structural homology models of the protein sequences were generated using the Swiss-Model server (https://swissmodel.expasy.org) (32), and PyMOL version 2.033 (40) was used to examine the molecular structures obtained and generate solvent-accessible electrostatic surfaces.

### Heterologous expression of HMFOs in E. coli.

The codifying DNA sequences of the HMFOs from *Methylovorus* sp. M668, P. nitroreducens, *Pseudomonas* sp. 11/12A, *Xanthobacter* sp. 126, and Bradyrhizobium arachidis (NCBI accession numbers WP_013440946, WP_024766380, WP_047529632, WP_024277017, and WP_092217059, respectively) were manually optimized for E. coli expression and synthesized by ATG:biosynthetics. The sequences were subcloned from the pGH vector into the pET23b(+) or pET28a(+) expression vector. C41(DE3) E. coli cells were used for expression. E. coli cells containing the expression vectors were grown overnight in LB broth supplemented with the corresponding antibiotic at 37°C under continuous shaking. These precultures were used to inoculate 1-liter amounts of LB (supplemented with the antibiotic) and the cultures grown for 3 to 4 h at 37°C under continuous shaking until reaching an optical density at 500 nm of 0.9. Cultures were induced with 0.1 mM isopropyl-β-d-thiogalactopyranoside, grown for 72 to 96 h at 16°C, and then harvested by centrifugation. Protein expression was monitored by sodium dodecyl sulfate-polyacrylamide gel electrophoresis (SDS-PAGE).

### Purification of recombinant HMFOs.

The bacterial pellets were resuspended in the corresponding lysis buffer (Table S3 in the supplemental material), and the mixtures were incubated with 0.1 mg/ml of DNase I and 2 mg/ml lysozyme for 45 min. The solutions were sonicated and centrifuged to eliminate insoluble debris.

*Metsp*HMFO and *Psesp*HMFO (untagged proteins) were purified in a ResourceQ 6-ml column (GE Healthcare) with a linear NaCl gradient (60 to 160 mM in 4 column volumes [CV]) in 50 mM Tris-HCl, pH 8.0, followed by a MonoQ 5/50 GL column in which the protein fractions were eluted with a NaCl linear gradient (0 to 70 mM in 10 CV) in 50 mM Tris-HCl, pH 8.0 (Table S3).

His-tagged fusion proteins (*Metsp*HMFO^His^ and *Pseni*HMFO^His^) were purified by affinity chromatography in a HiTrap IMAC FF column (GE Healthcare) with a linear imidazole gradient (20 m to 300 mM in 6 CV) in 500 mM NaCl, 20 mM Tris-HCl, pH 7.5, followed by anionic exchange chromatography with a ResourceQ column (GE Healthcare) with an NaCl linear gradient (0 to 500 mM in 5 CV) in 50 mM Tris-HCl, pH 7.5 (Table S3).

The purified HMFOs were dialyzed against 50 mM Tris-HCl, pH 7.0, and stored at −80°C, being stable for several months.

### Spectroscopic measurements.

The UV-visible spectra of purified HMFOs were recorded between 250 and 700 nm in a Cary4000 spectrophotometer. Their extinction coefficients were calculated by heat denaturation (41) and estimation of the free FAD released (ε_band I_ = 11,300 mM^−1^ cm^−1^) (42).

Far-UV CD spectra of HMFOs (5 μM) were recorded in a Jasco J-815 spectropolarimeter at 25°C in 5 mM Tris-HCl, pH 7.0, with a 0.1-cm path length. The spectra were analyzed with the CDPro programs SELCON3 (43), CDSSTR (44), and CONTINLL (45) to determine the secondary structure.

### MW and pI determination.

MW was analyzed by MALDI-TOF mass spectrometry at the Proteomics and Genomics Facility of CIB (CSIC), a member of the ProteoRed-ISCIII network. The experiments were performed in an Autoflex III MALDI–tandem-TOF instrument (Bruker Daltonics, Bremen, Germany) with a smart beam laser. Samples dissolved in 50 mM Tris-HCl, pH 7.0, were mixed with the matrix solution (3.5-dimethoxy-4-hydroxycinnamic acid dissolved in a mixture of acetonitrile–01% trifluoroacetic acid [1:2, vol/vol]). External calibration was performed using bovine albumin (Sigma), covering the range from 20,000 to 70,000 Da.

The pI was determined by 2-dimensional (2-D) electrophoresis. The first dimension was run on immobilized pH gradient strips (pH 3 to 10, linear, 7 cm) (Bio-Rad), and the second dimension was run on 12% SDS–PAGE gels. Protein bands were stained with a colloidal blue staining kit (Invitrogen).

### pH and H_2_O_2_ stability.

The pH and H_2_O_2_ stabilities were estimated by incubating the enzymes (5 to 40 μM) in 100 mM Britton and Robinson (B&R) buffer at different pHs (pH 4.0 to 9.0) or in the presence of different H_2_O_2_ concentrations (0.5 to 7.8 mM) in 50 mM Tris-HCl, pH 7.0. Residual activities were estimated by following the oxidation of saturating concentrations of vanillyl alcohol (3 mM) in 50 mM Tris-HCl, pH 7.0, at 25°C, immediately after mixing and after 24 h, 48 h, and 72 h of incubation at 25°C. For each enzyme, the highest activity after mixing was taken as 100% activity, and the percentages of residual activity at the different times and conditions were calculated according to this maximal value.

### Thermal stability.

The melting temperatures of the enzymes were analyzed by the ThermoFAD method (26). The increase in fluorescence due to FAD released as a consequence of enzyme unfolding was monitored with a real-time PCR thermocycler iQ5 (Bio-Rad). Denaturation curves were fitted to equation 1, describing a two-step thermal denaturation equilibrium:

(1) $$\begin{array}{c} F=\frac{F_{N}+m_{N}T+(F_{D}+m_{D}T)e^{-\Delta G/\mathrm{RT}}}{1+e^{-\Delta G/\mathrm{RT}}} \end{array}$$

where $\Delta G=\Delta H\left[1-\left(T/T_{m}\right)\right]-\Delta \mathrm{Cp}\left[\left(T_{m}-T\right)+T\;\ln\left(T/T_{m}\right)\right]$, *F_N_* and *F_D_* are the fluorescence signals of the native and denatured states, *m_N_* and *m_D_* are the slopes that describe their dependences with temperature, *T* and *T_m_* are the temperature and the melting temperature, respectively, *R* is the universal gas constant, and Δ*G*, Δ*H*, and Δ*C_p_* are the free energy, enthalpy, and specific heat of denaturation, respectively.

### Optimal pH and temperature.

The optimal temperatures and pHs for vanillyl alcohol oxidation by HMFOs were determined by measuring the oxidation of saturating concentrations of the substrate (3 mM) in 50 mM Tris-HCl buffer, pH 7.0, at different temperatures between 4°C and 70°C or in 100 mM B&R buffer in the range of pHs from 2.0 to 12.0. It was observed that the UV-visible spectra of vanillyl alcohol and vanillin changed with pH (Fig. S5a and b) (46). Thus, in the case of the optimal pH, the activity was measured using the extinction coefficient for vanillyl alcohol oxidation to vanillin calculated for each pH (Fig. S5c).

### Steady-state kinetics.

Enzymatic activity was estimated by following the production of vanillin upon oxidation of vanillyl alcohol at 25°C in 50 mM Tris-HCl at the indicated pH in each case, using the corresponding extinction coefficient for vanillyl alcohol oxidation to vanillin (Fig. S5c).

Steady-state kinetic parameters for HMF and DFF oxidation by HMFOs were calculated by monitoring the production of H_2_O_2_ during oxidation of the different substrates, using an HRP-coupled assay with AmplexRed as the final substrate in 50 mM Tris-HCl, pH 7.0, as previously described (16).

The oxidation of FFCA was measured in end-time mode by incubating different concentrations of FFCA (0.4 to 6 mM) with the enzymes (1 μM) at 25°C in 50 mM Tris-HCl buffer, pH 7.0. After 48 h, reactions were stopped by the addition of 1 M HCl up to pH 2 to 3, and the products were quantified by HPLC as described below.

In all cases, kinetic parameters were determined by fitting the initial reaction rates at different alcohol or aldehyde concentrations to the Michaelis-Menten equation (equation 2) or to the Hill equation (equation 3) using SigmaPlot software.

(2) $$\frac{v_{0}}{\left[E\right]}=\frac{k_{\text{cat}}[S]}{K_{m}+[S]}$$

(3) $$\frac{v_{0}}{\left[E\right]}=\frac{k_{\text{cat}}{\left[S\right]}^{n}}{K_{m}^{n}+{\left[S\right]}^{n}}$$

where *E* and *S* are the concentrations of enzyme and substrate, respectively, *k*_cat_ is the catalytic constant, *K_m_* is the Michaelis-Menten constant, and *n* is the Hill coefficient.

### HMF transformations.

Long-term HMF oxidation by HMFOs was assayed using 2.5 to 5 μM enzyme and 1.5 mM HMF in 50 mM Tris-HCl, at the pHs and temperatures indicated in Results for each experiment, under continuous shaking (300 rpm). Samples were taken at different times, and reactions were stopped by HCl addition. The products of the reactions were analyzed by HPLC, using an ion exchange Supelcogel C-610H column (300 by 7.8 mm, 9-μm particle size; Sigma). Compounds were eluted with 5 mM H_2_SO_4_ at a flow rate of 0.75 ml/min and 30°C. Under these conditions, the retention times of FDCA, FFCA, HMF, and DFF were 15.5, 21.3, 30.2, and 37.3 min, respectively. Detection of the different compounds was done at 264 nm, and their quantification was performed using the corresponding calibration curves (16). Controls consisting of the substrates in the absence of enzyme were treated in the same way to monitor eventual spontaneous oxidation.

Residual activity of HMFOs along the reactions was measured just after taking aliquots, before the addition of HCl, by monitoring their activities against vanillyl alcohol. The activity decay as a function of time was calculated from equation 4, allowing the estimation of the half-life (equation 5):

(4) HMFO activity (%)=HMFOact0 e(−λ×t)

(5) $$t_{1/2}=\frac{\text{ln}\left(2\right)}{\lambda }$$

where λ is the activity decay constant and *t*_1/2_ is the half-life.

### Data availability.

All data are included here and in the supplemental material.

## Supplementary Material

Supplemental file 1
