# Supplementary material for: Screening and Evaluation of New Hydroxymethylfurfural Oxidases for Furandicarboxylic Acid Production
Source: Appl Environ Microbiol. 2020 Aug 3;86(16):e00842-20. doi: 10.1128/AEM.00842-20 (PMC7414962; doi:10.1128/AEM.00842-20)
Supplement: Supplemental file 1 [file AEM.00842-20-s0001.pdf]

SUPPLEMENTAL MATERIAL

Screening and Evaluation of New Hydroxymethylfurfural Oxidases for Furandicarboxylic Acid Production

Mario Viñambres,[†] Marta Espada,[†] Angel T. Martínez,[#] Ana Serrano[#]

[1]Centro de Investigaciones Biológicas Margarita Salas, CSIC, Ramiro de Maeztu 9, E-28040 Madrid, Spain

[#]Corresponding authors: anaserra@cib.csic.es and ATMartinez@cib.csic.es
[†]Mario Viñambres and Marta Espada contributed equally to this study and the authors order corresponds to their successive contribution to the work

Supplemental material includes: Inventory of HMFO sequences from GenBank (**Table S1**), Nucleotide sequences of five *hmfo* genes (**Table S2**), Conditions for purification of the different HMFOs (**Table S3**), Secondary structure elements of the different HMFOs **(Table S4**), Sequence alignment of HMFOs (**Fig. S1**), SDS-PAGE along the purification of HMFOs (**Fig. S2**), Spectroscopic properties of HMFOs (**Fig. S3**), Residual activities during HMF oxidation under different conditions (**Fig. S4**), Changes in spectroscopy properties of vanillyl alcohol and vanillin with pH (**Fig. S5**).

Table S1. Information on the protein sequences obtained (identity >45%) after BLASTing *Metsp*HMFO against the GenBank database, with the five enzymes optimized for *E. coli* production in bold.

ID number	Species	Score	E-value	Identity (%)	Length (aa)	$MW_{theoretical}$	$pI_{theoretical}$
**WP_013440946**	***Methylovorus* sp. MP688**	**--**	**--**	**--**	**531**	**57014.41**	**5.87**
WP_015829137	*Methylovorus glucosotrophus*	1054	0.0	99.06	531	56965.34	5.92
WP_024929608	*Methylophilus* sp. OH31	749	0.0	70.32	531	57441.61	5.02
WP_019881601	*Methylophilus*	751	0.0	70.13	531	57517.62	4.91
WP_047514482	*Methylophilus* sp. Q8	750	0.0	70.13	531	57516.68	4.98
WP_131182903	*Pseudomonas* sp.	641	0.0	63.57	528	57373.83	5.85
WP_122949865	*Burkholderia lata*	630	0.0	63.53	533	56690.08	6.37
WP_054910186	*Pseudomonas*	647	0.0	63.52	531	57081.33	5.81
**WP_024766380**	***Pseudomonas nitroreducens***	**647**	**0.0**	**63.52**	**531**	**57084.20**	**5.65**
WP_084358398	*Pseudomonas nitroreducens*	646	0.0	63.52	531	57154.34	5.72
WP_065086293	*Pseudomonas* sp. AU12215	644	0.0	63.52	531	57128.26	5.65
WP_131187983	*Pseudomonadaeae bacterium* P30C	638	0.0	63.38	528	57312.70	5.73
**WP_047529632**	***Pseudomonas* sp. 11/12**	**660**	**0.0**	**62.85**	**530**	**57130.38**	**5.68**
WP_090462207	*Pseudomonas mohnii*	659	0.0	62.66	530	57107.41	5.68
WP_105697938	*Pseudomonas poae*	659	0.0	62.48	530	57189.27	6.09
WP_048372898	*Pseudomonas helleri*	624	0.0	61.54	530	57271.30	5.53
WP_110950867	*Pseudomonas bohemica*	622	0.0	60.98	530	57137.32	5.69
OYU07719	*Pseudomonas* sp. PGPPP1	638	0.0	60.23	530	57209.04	5.97
**WP_092217059**	***Bradyrhizobium arachidis***	**429**	**2e-141**	**47.00**	**565**	**61033.90**	**9.12**
WP_012331246	*Methylobacterium* sp. 4-46	426	3e-140	46.82	570	60407.96	9.41
**WP_029556649**	***Xanthobacter* sp. 91**	**432**	**1e-142**	**46.81**	**562**	**59717.16**	**7.69**
WP_018262292	*Methylobacterium* sp. WSM2598	424	1e-139	46.64	570	60393.93	9.41
WP_029002663	*Azorhizobium doebereinerae*	452	1e-150	46.63	568	59855.51	8.86

WP_131320784	*Burkholderia* sp. WK1.1f	447	1e-148	46.58	582	62871.00	8.92
WP_128925190	*Bradyrhizobium guangxiense*	434	2e-143	46.52	565	61195.13	8.51
WP_024277017	*Xanthobacter* sp. 126	427	9e-141	46.45	565	60117.48	6.48
WP_063984192	*Bradyrhizobium* sp.	421	2e-138	46.41	565	60808.66	8.75
WP_128965697	*Bradyrhizobium guangdongense*	440	9e-146	46.34	565	61229.21	9.28
WP_057744137	*Bradyrhizobium manausense*	423	4e-139	46.34	565	61423.24	8.90
WP_132475546	*Paracandidimonas soli*	457	2e-152	46.15	566	62476.45	8.78
WP_038934019	*Bradyrhizobium japonicum*	430	6e-142	46.06	565	61068.90	8.92
WP_060849362	*Methylobacterium aquaticum*	405	6e-132	46.05	571	60849.36	8.23
WP_048436760	*Methylobacterium platani*	404	1e-131	46.05	571	60903.45	8.23
WP_039150218	*Bradyrhizobium japonicum*	426	2e-140	45.88	565	61129.05	9.18
WP_028149427	*Bradyrhizobium japonicum*	424	9e-140	45.88	565	61004.82	9.08
WP_071915236	*Bradyrhizobium japonicum*	417	6e-137	45.88	565	60782.50	8.24
WP_048427955	*Methylobacterium platani*	403	3e-131	45.87	571	60889.43	8.23
WP_020066156	*Paraburkholderia caledonica*	442	2e-146	45.86	582	62868.92	8.81
WP_015931284	*Methylobacterium nodulans*	403	3e-131	45.80	573	60854.53	8.98
WP_086651868	*Acetobacter cibinogensis*	410	1e-134	45.79	540	57775.10	5.58
SFF24619	*Methylobacterium* sp. yr596	402	5e-131	45.69	573	61194.84	8.55
WP_091887066	*Methylobacterium* sp.	402	5e-131	45.69	571	60907.46	8.23

Table S2. Nucleotide sequences of the five *hmfo* genes optimized for *E. coli* expression (FASTA format).

```
>METSP_WP013440946
CATATGACTGATACGATTTTTGACTACGTGATTGTTGGCGGTGGCACGGCGGGTAGCGTTCTGGCCAA
CCGTCTGTCCGCCCGTCCGGAGAATCGCGTGTTGCTGATTGAGGCCGGTATTGATACCCCGGAAAACA
ATATTCCGCCGGAGATCCACGATGGCCTGCGCCCGTGGCTGCCGCGTCTGAGCGGTGATAAGTTCTTT
TGGCCGAATCTGACCATCCACCGTGCCGCGGAACACCCGGGTATCACGCGCGAGCCGCAGTTCTATGA
ACAAGGCCGTCTGCTGGGCGGTGGTAGCAGCGTGAACATGGTCGTTTCTAACCGTGGTCTGCCTCGCG
ACTATGACGAATGGCAGGCACTGGGCGCAGATGGTTGGGATTGGCAGGGTGTTCTGCCGTACTTCATC
AAGACCGAGCGTGACGCGGACTACGGTGACGACCCGTTGCATGGCAATGCGGGTCCGATTCCGATCGG
TCGCGTCGATTCGCGTCACTGGAGCGACTTCACGGTGGCGGCAACCCAAGCTCTGGAAGCGGCTGGCC
TGCCGAACATTCACGACCAAAACGCACGTTTTGATGACGGTTACTTCCCACCGGCATTTACGTTGAAA
GGTGAAGAGCGCTTCAGCGCCGCACGCGGTTATCTGGATGCGAGCGTCCGTGTGCGTCCGAACCTGAG
CCTGTGGACTGAGAGCCGTGTCCTGAAGCTGCTGACCACTGGCAATGCAATCACCGGTGTGAGCGTGC
TGCGTGGTCGCGAAACCCTGCAAGTTCAAGCGCGCGAGGTCATCCTGACCGCCGGTGCGTTGCAAAGC
CCAGCGATTCTGTTGCGCACCGGCATCGGCCCTGCGGCGGATCTGCACGCACTGGGTATTCCTGTTCT
GGCAGACCGTCCGGGTGTTGGTCGCAATCTGTGGGAGCACAGCTCTATCGGTGTGGTTGCCCCGCTGA
CCGAGCAGGCACGTGCAGACGCCAGCACGGGTAAAGCCGGCTCTCGCCATCAACTGGGTATCCGTGCG
TCGTCCGGCGTAGATCCGGCGACGCCTAGCGACCTGTTTCTGCATATCGGTGCTGATCCAGTCAGCGG
TCTGGCAAGCGCTGTGTTCTGGGTGAACAAGCCAAGCTCCACCGGCTGGCTGAAGCTGAAGGACGCGG
ACCCGTTTAGCTACCCGGACGTAGACTTCAATCTGCTGAGCGATCCGCGCGACTTGGGTCGTCTGAAA
GCGGGCCTGCGTCTGATCACCCATTACTTCGCAGCGCCGTCCCTGGCGAAATATGGTTTGGCGCTGGC
ATTGAGCCGTTTTGCGGCACCGCAGCCGGGTGGTCCGCTGCTGAACGACCTGTTGCAGGACGAAGCCG
CCCTGGAACGCTATTTGCGTACGAACGTCGGCGGTGTTTGGCATGCGAGCGGCACGGCGCGTATCGGC
CGTGCGGATGATTCCCAGGCTGTTGTCGATAAAGCGGGTCGTGTGTACGGCGTCACCGGCCTGCGTGT
TGCGGACGCAAGCATTATGCCGACCGTTCCGACCGCCAATACCAATCTGCCGACGCTGATGCTGGCTG
AGAAAATTGCGGATGCGATTCTGACCCAGGCTTAAGGATCC

>PSESP_WP047529632
ATATGAGCATTGAACATTATGATTATATTATTGTGGGCGGCGGCAGCGCGGGCAGCGTGATTGCGAAC
CGCCTGAGCGCGCGCGCGGATCGCCGCGTGCTGCTGATTGAAGCGGGCGCGGATACCCCGGATAGCAA
CACCCCGGCGGAAATTCTGGATGGCCTGCAGCCGTGGCGCCCGCGCCTGGCGGGCGAACGCTATTTTT
GGCCGGATCTGACCATTCTGCGCGCGAGCGAACATCCGGATATTCCGCGCGATGCGCAGGTGTATGAA
CAGGGCCGCATTCTGGGCGGCGGCAGCAGCGTGAACATGATGGTGGCGAACCGCGGCCTGCCGCGCGA
TTATGATGAATGGGCGCAGCAGGGCGCGGATGGCTGGAACTGGCAGGGCGTGCTGCCGTATTTTCGCA
AACTGGAACGCGATCTGGATTTTGGCCAGAGCGATCCGGAACAGCATGGCCATGAAGGCCCGCTGCCG
ATTGCGCGCGTGGATAGCCGCTATTGGGGCAGCTTTACCCGCGCGAGCGCGCAGGCGCTGCAGGCGCT
GGGCCTGAACAACATTGTGGATCAGAACGGCCGCTTTGAAGATGGCTATTTTGCGCCGACCGTGGCGA
TTGAACATGATCAGCGCGTGAGCAGCGCGCGCGCGTATCTGACCCCGCAGGTGCGCGCGCGCAGCAAC
CTGACCCTGTGGACCGATACCCCGGTGCGCCAGCTGATTCTGAGCGGCAACCGCGTGACCGGCGTGCA
GGTGCTGCGCGATGGCAAACGCCTGGAAGTGAGCGGCAACGAAGTGATTCTGACCGCGGGCGCGCTGC
AGAGCCCGGCGTTTCTGCTGCGCGCGGGCATTGGCCCGGCGGATCAGCTGCGCGATCTGGGCATTGCG
GTGGTGGCGGATCGCAGCGGCGTGGGCCGCAACCTGTGGGATCATAGCAGCATTGGCCTGACCGCGAC
CCTGCATGATCCGCTGGCGCCGAGCGATGAACCGGGCGCGGCGCATCTGCTGGCGGCGCGCGTGAGCA
GCGGCGTGGCGCCGGGCGTGCCGGCGGATCTGTATCTGGGCATTGCGCCGAACGCGGCGCTGGGCACC
GCGAGCGCGGTGCTGTGGGTGAACAAACCGAGCAGCACCGGCCATCTGCGCCTGCGCAGCGTGGACCC
GATGGATTATCCGGCGGTGGAATTTAACCTGCTGAGCGATCGCCGCGATCTGGATCGCCTGCGCGAAG
GCCTGCGCGTGCTGGCGCGCGTGTTTGCGGACCCGGCGGTGGCGGCGTTTAGCGGCCCGCCGCGCCTG
AGCCGCTTTGCGCAGGCGAGCACCGATGGCCCGCTGCTGAGCGAACTGCTGGCGAACGATGTGGCGCT
GGAACGCTATCTGCGCACCCATGTGGGCGGCGTGTGGCATGCGAGCGGCACCTGCCGCATTGGCCGCC
CGGATGATCCGCTGGCGGTGGTGGATCATGCGGGCAAAGTGTATGGCGTGCAGGGCCTGCGCGTGGCG
GATGCGAGCGTGATGCCGAGCATTCCGGCGGCGAACACCAACCTGCCGACCCTGATGATTGCGGAAAA
AATTGCGGATGCGATTCTGACCGATGCGTAAGGATCC
```

>PSENI_WP024766380
CATATGACCACCCCGCGTTACGACCACATCATCGTTGGTGGTGGTTCTGCGGGTTCTGTTCTGGCGTC
TCGTCTGTCTGCGGACCCGCAGCGTCACGTTCTGCTGATCGAAGCGGGTCCGGACACCCCGCCGCACG
CGACCCCGGCGGAAATCCTGGACGGTCACAACCCGTTCCGTCTGCGTTGGAACTGCGTGACCGTTACT
TCTGGCCGGACCTGTCTGTTCGTCACGGTGCGCAGCCGGAAGGTATCGAACGTCCGGAACGTTACCTG
GAACAGGCGCGTGTTCTGGGTGGTGGTTCTTCTGTTAACGTTCTGGTTGCGAACCGTGGTCTGCCGCG
TGACTACGACCAGTGGGCGGCGCTGGGTGCGGAAGGTTGGGGTTGGGACGACGTTCTGCCGTACTTCC
GTAAACTGGAACGTGACACCGACTACGCGGGTCCGCTGCACGGTCACGACGGTCCGATCCCGATCTCT
CGTGTTTCTACCCGTGACTGGACCCCGTTCACCCGTTCTGTTGTTTCTGCGCTGCAGGCGGAAGGTCT
GCGTGACATCGGTGACCAGAACGGTGTTTTCGACGACGGTTACTTCGCGCCGGCGGTTAACCTGGAAC
ACGGTCAGCGTGTTTCTGCGGCGCGTGGTTACCTGGACGAAGCGGTTCGTGCGCGTCCGAACCTGACC
CTGTGGACCGACTCTCAGGTTCTGGGTCTGCAGCGTGAAGGTACCCGTATCACCGGTGTTGAACTGCA
GCGTAACGGTGAACACGTTACCGTTGGTGCGGGTGAAGTTATCCTGGCGGCGGGTGCGCTGCAGTCTC
CGGCGTTCCTGCTGCGTTCTGGTATCGGTCCGGCGTCTCAGCTGCAGGAACTGGGTATCGAAGTTATC
GCGGACCGTCCGGGTGTTGGTGGTAACCTGTGGGAACACTCTTCTCTGGGTACCCTGGCGACCCTGGG
TGACGCGGCGCGTTCTGACGCGGTTCTGGCGCGTCCGGGTACCTCTCACCAGCTGGGTATCCGTGTTT
CTTCTGGTGTTGACCCGGACACCCCGTCTGACCTGTACCTGGCGATCGGTGCGGACGCGGAACGTGGT
GTTGCGCACGCGCTGTTCTGGATCAACAAACCGTCTTCTTCTGGTCGTCTGACCCTGCGTGACCGTGA
CCCGGCGTCTCCGCCGCACGTTGACTTCAACCTGCTGTCTGACCCGCGTGACCTGCAGCGTGCGCGTG
TTGCGCTGCGTACCATCCAGCGTCTGTTCAAACACCCGGTTCTGGCGCGTTACGAACTGCAGCTGGCG
CTGACCCCGTTCGCGGTTCCGCAGCCGGGTGGTCCGAAACTGGAAACCCTGCTGGCGGACGACACCGC
GCTGGAAGCGTACCTGCGTCAGCACATCGGTGGTGTTTGGCACCCGTCTGGTACCTGCCGTATCGGTA
CCGCGGACGACCCGCAGGCGGTTGTTGACTCTGCGGGTCGTGTTCACGGTGTTGAAGGTCTGCGTGTT
GCGGACGCGTCTATCATCCCGGTTATCCCGACCGCGAACACCAACCTGCCGACCCTGATGCTGGCGGA
AAAAATCGCGGACGCGATCCTGGGTGGTGCGTAAGGATCC

> XANSP_WP024277017
CATATGCCGGACATGCCGTTCTTCGACACCGTTATCGTTGGTGGTGGTTCTGCGGGTTGCGTTATGGC
GAACCGTCTGTCTGCGGACCCGGCGCACTCTGTTCTGCTGATCGAAGCGGGTGAAGACACCCCGCCGG
GTGCGGTTCCGGACGCGATCGCGGACTCTTACCCGATGGGTCTGTTCCACGGTGACCGTTTCGTTTGG
CCGGGTCTGTCTGCGTTCACCGCGCACCGTCCGGACGGTACCCGTGGTTCTCGTGCGTACGAACAGGG
TCGTGTTATGGGTGGTGGTTCTTCTATCAACGTTCAGTCTGCGAACCGTGGTCTGCCGCGTGACTACG
ACGAATGGGCGGCGCTGGGTGCGGCGGGTTGGGGTTGGGACGACGTTCTGCCGTTCTTCATCAAACTG
GAACGTGACCTGGACTGCGACGGTCCGCTGCACGGTCGTGACGGTCCGATCCCGATCCGTCGTATCCT
GCCGCCGGGTTGGCCGCCGTTCGCGGAAGCGGCGACCTCTGCGTTCGCGGCGTCTGGTCTGCCGCGTC
GTCTGGACCAGAACGGTGAATTCGAAGACGGTGTTTTCCCGCCGGCGTTCAACAACGAAAACGACCGT
CGTGTTTCTGCGGCGATCGGTTACCTGGACGCGGCGACCCGTGCGCGTCCGAACCTGGCGATCTGGCC
GCGTACCCAGGTTCTGCGTCTGCGTATGGACGGTGCGCGTGCGGCGGGTGTTCAGGTTGAACGTGACG
GTGTTGTTACCGAAGTTGCGGCGGGTCGTGTTGTTGTTACCGCGGGTGCGCTGCAGACCCCGGCGATC
CTGATGCGTGCGGGTATCGGTGACGGTGTTGCGCTGGCGGCGCTGGGTCTGCCGGTTGTTATCCACCG
TCCGGCGGTTGGTCGTAACCTGCGTGACCACCCGACCCTGACCTTCTGCCAGTACCTGCCGGAACGTC
TGCGTCTGCCGATGTCTTACCGTCGTGCGTCTTTCGCGGCGCTGCGTTTCTCTTCTGGTGTTGACGGT
GGTGAAGCGTCTGACCTGTACGCGACCGCGTCTGCGCGTGCGGGTTGGCACGGTCTGGGTCGTCGTCT
GGGTCTGTACTTCCTGTGGTGCAACCGTCCGAAATCTTCTGGTTCTGTTCGTCTGGTTTCTGCGGACC
CGCGTGCGTACCCGCAGGCGGACCTGAACCTGCTGGACGACCCGTCTGACCTGGCGCGTATGAAAGCG
GGTGTTCGTACCCTGTTCTCTCTGCTGGTTTGCGAAGCGCTGAACCCGGACGCGGGTGACGTTTTCCC
GGCGGCGTTCTCTCCGTTCGTTAAACGTCTGTCTCGTGTTACCCGTACCAACGCGCTGCTGGCGGAAG
CGCTGGGTGCGCTGCTGGACGTTCCGGCGGCGCTGCGTCAGACCGCGCTGAAACGTTTCATGTCTGGT
GGTGTTTCTCTGGCGGAAATCGTTGCGGACGAAGCGCGTCTGGAAGCGTTCGTTCGTTCTTCTACCTT
CGGTGTTTGGCACGCGTCTGGTACCTGCCGTCTGGGTGCGCCGGACGACCCGGAAGCGGTTCTGGACC
CGTCTGGTCGTGTTATCGGTTCTGAAAACCTGTACGTTGCGGACGCGTCTGCGATGCCGCGTCTGCCG
TCTGCGAACACCAACATCCCGGTTCTGATGATCGCGGAAAAAATCGCGGCGGGTCTGGCGCGTGCGTA
AGGATCC

>BRAAR_WP092217059
CATATGGATGGCCTGCATTTTGATGATGTGATTGTGGGCGGCGGCAGCGCGGGCTGCGTGCTGGCGAA
CCGCCTGAGCGCGGATGCGCGCCGCCATGTGCTGCTGATTGAAGCGGGCATGGATACCCCGCCGGAAG
CGACCCCGGCGGAAATTCTGGATAGCTATCCGATGCCGCTGTTTTTTGGCGATAAATATATTTGGCCG
GGCCTGAGCGCGGCGGCGGGCCGCAACGCGGAAGGCAAACCGGTGGTGCGCGCGTATGAACAGGGCCG
CGTGATGGGCGGCGGCAGCAGCATTAACGTGCAGAGCGCGAACCGCGGCCTGCCGCGCGATTATGATG
AATGGCATGAACTGGGCGCGCGCGGCTGGGGCTGGACCGATGTGCTGCCGTATTTTCTGAAACTGGAA
ACCGATCTGGATTTTGGCGGCGCGCTGCATGGCCGCGGCGGCCCGATTCCGATTCGCCGCATTGCGCG
CGAAGCGATGCCGGCGTTTGGCCGCGCGGCGGGCGAAGCGCTGAGCGCGACCGGCCTGCCGTTTCGCA
AAGATCAGAACGCGGAATTTGAAGATGGCATTTTTCCGCCGGCGTTTAGCAACCGCAACGATCGCCGC
GTGAGCACCGCGGCGGGCTATCTGGATGCGGCGACCCGCGCGCGCCCGAACCTGACCATTTGGGCGGG
CAGCGAAGTGGAACAGCTGCAGCTGGATGGCCGCCGCGCGCGCAGCGTGGTGGTGGCGCGCGGCGGCC
AGAAACTGACCGTGGGCGCGGGCCGCGTGATTCTGACCGCGGGCGCGCTGCAGAGCCCGGCGATTCTG
ATGCGCGCGGGCATTGGCCCGGGCCCGGCGCTGCAGGCGCTGGGCATTCCGGTGGCGATTGATCTGCC
GGGCGTGGGCGGCAACCTGCGCGATCATCCGGCGCTGACCTTTTGCCAGTATCTGCCGCGCCGCCTGC
GCCTGCCGCTGGCGCGCCGCCGCCCGAACTTTACCGCGATGCGCTTTAGCAGCGGCCAGCCGGGCTGC
GATCCGAGCGATATGTATATTACCGCGAGCGCGCGCGGCGGCTGGCATGCGCTGGGCACCCGCCTGGG
CCTGTATTTTCTGTGGTGCAACCGCCCGTATAGCAGCGGCAGCCTGACCCTGGCGAGCCAGGACCCGA
AAGCGTATCCGGTGGTGGATTTTAACCTGCTGAGCGATGAACGCGATGTGGAACGCCTGGTGAGCGCG
GTGCGCCTGCTGGCGCGCCTGGTGGTGCATCCGCAGCTGAACCCGGAAGCGGGCGATTTTTTTCCGGC
GAGCTATAGCCCGCGCATTAAACGCCTGAGCCGCTATGGCACCGCGAACCGCATTGTGGCGAGCATTC
TGGGCCCGATGCTGGATGTGCCGGCGGGCCTGCGCCAGCTGCTGATTCGCCTGGTGCTGCTGAACGGC
ACCGCGTTTCAGGCGACCCTGGCGGATGAACGCGCGCTGGAAGCGTTTGTGCGCCAGAGCGTGTTTGG
CGTGTGGCATCCGGTGGGCACCTGCCGCATGGGCGATCCGGCGGATCGCATGGCGGTGGTGGACCCGG
GCGGCCGCGTGATTGGCAGCGAAAACGTGTATGTGGCGGATGCGAGCATTATGCCGCGCCTGCCGACC
GCGAACACCAACATTCCGGTGATTATGGCGGCGGAAAAAATTAGCGATGCGCTGCTGCGCCATCATTA
AGGATCC

Table S3. Summary of the conditions used for purification of the different HMFOs.

Enzyme	Cloning vector	Lysis buffer	Chromatographic steps	Elution gradient
*Metsp*HMFO	pET23b(+)	10 mM EDTA<br>5 mM DTT<br>20 mM Tris/HCl<br>pH 8.0	ResourceQ (5 mL)<br>MonoQ (1 mL)	NaCl: 60 – 160 mM (4 CV)<br>NaCl: 0 – 70 mM (10 CV)
	pET28a(+)	20 mM imidazole<br>300 mM NaCl<br>20 mM Tris/HCl<br>pH 8.0	HiTrap IMAC FF (5 mL)<br>ResourceQ (5 mL)	Imidazole: 20 – 300 mM (6 CV)<br>NaCl: 0 – 500 mM (5 CV)
*Psesp*HMFO	pET23b(+)	10 mM EDTA<br>5 mM DTT<br>20 mM Tris/HCl<br>pH 8.0	ResourceQ (5 mL)<br>MonoQ (1 mL)	NaCl: 60 – 160 mM (4 CV)<br>NaCl: 0 – 70 mM (10 CV)
*Pseni*HMFO	pET28a(+)	20 mM imidazole<br>300 mM NaCl<br>20 mM Tris/HCl<br>pH 8.0	HiTrap IMAC FF (5 mL)<br>ResourceQ (5 mL)	Imidazole: 20 – 300 mM (6 CV)<br>NaCl: 0 – 500 mM (5 CV)

Table S4. Percentages of secondary structure from CD spectra of the different HMFOs.

	α-helix	β-sheet	turn	unordered
*Metsp*HMFO	37 ± 3	17 ± 8	20 ± 2	26 ± 9
*Metsp*HMFO^{His}	27 ± 9	14 ± 2	24 ± 3	37 ± 7
*Psesp*HMFO	47 ± 9	11 ± 8	22 ± 7	20 ± 8
*Pseni*HMFO^{His}	27 ± 6	20 ± 3	23 ± 3	31 ± 4

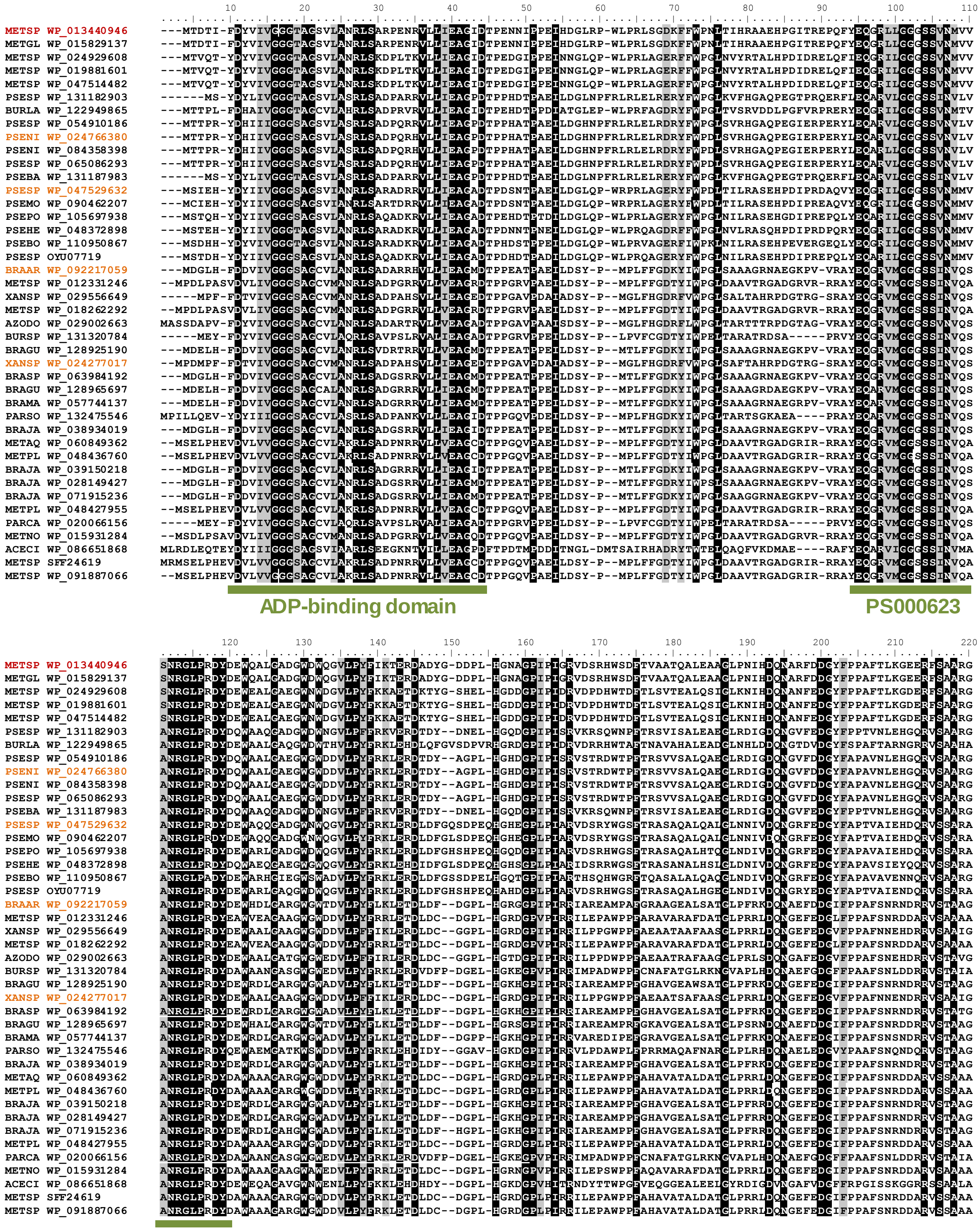

Fig. S1. Multiple alignment of 41 HMFO-like sequences from GeneBank screening, together with the *Metsp*HMFO sequence (WP_013440946, red). The *Pseni*HMFO (WP_024766380), *Psesp*HMFO (WP_047529632), *Braar*HMFO (WP_092217059) and *Xansp*HMFO (WP_029556649) sequences optimized for *E. coli* production are shown in orange. Residues in black and grey have 100% identity and similarity, respectively. Last line shows: i) conserved motifs in GMC oxidoreductases (ADP-binding, PS000623 and PS000624, green); and ii) two catalytic residues (His467 and Asn511) in *Metsp*HMFO (red stars).

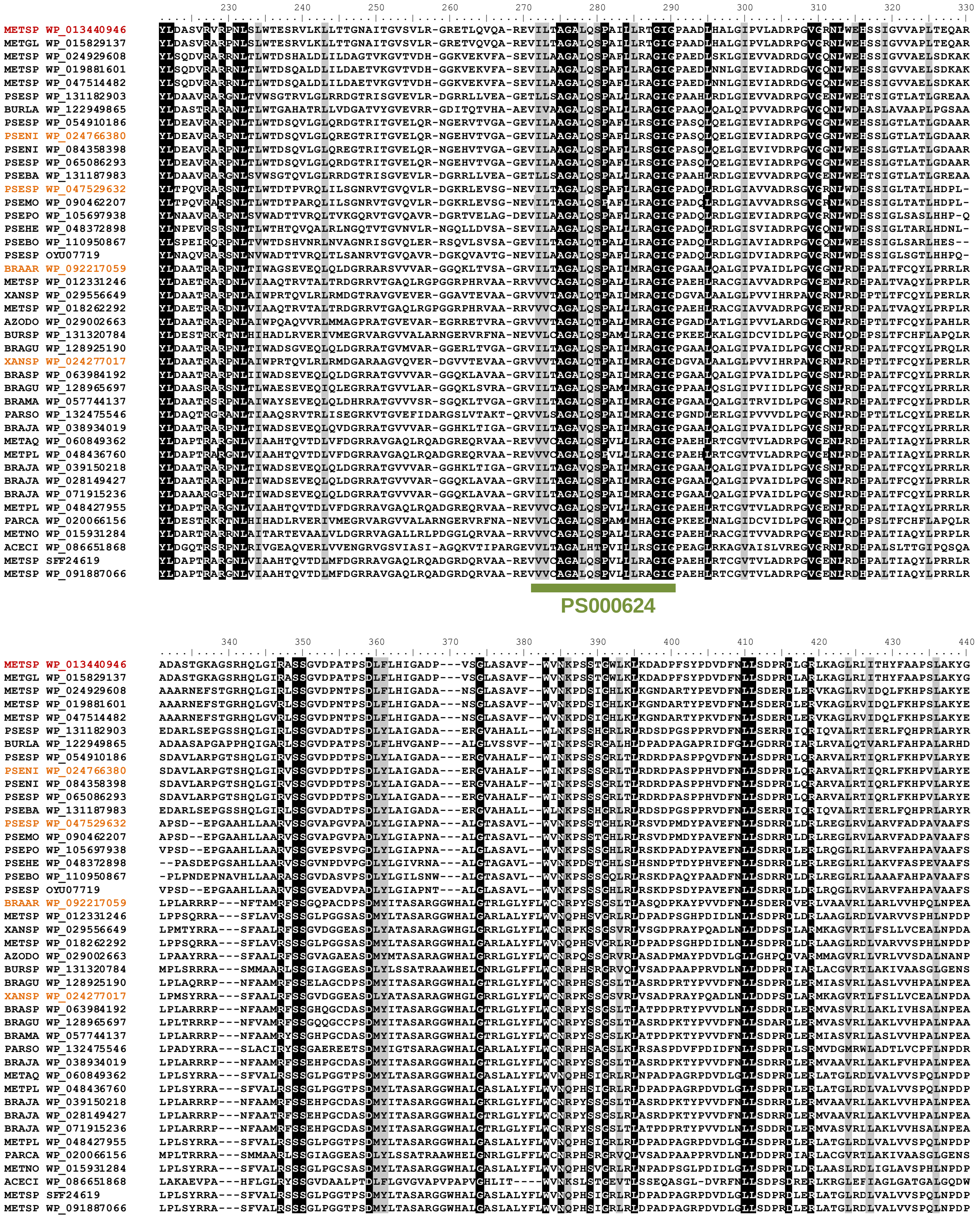

Fig. S1. (cont.)

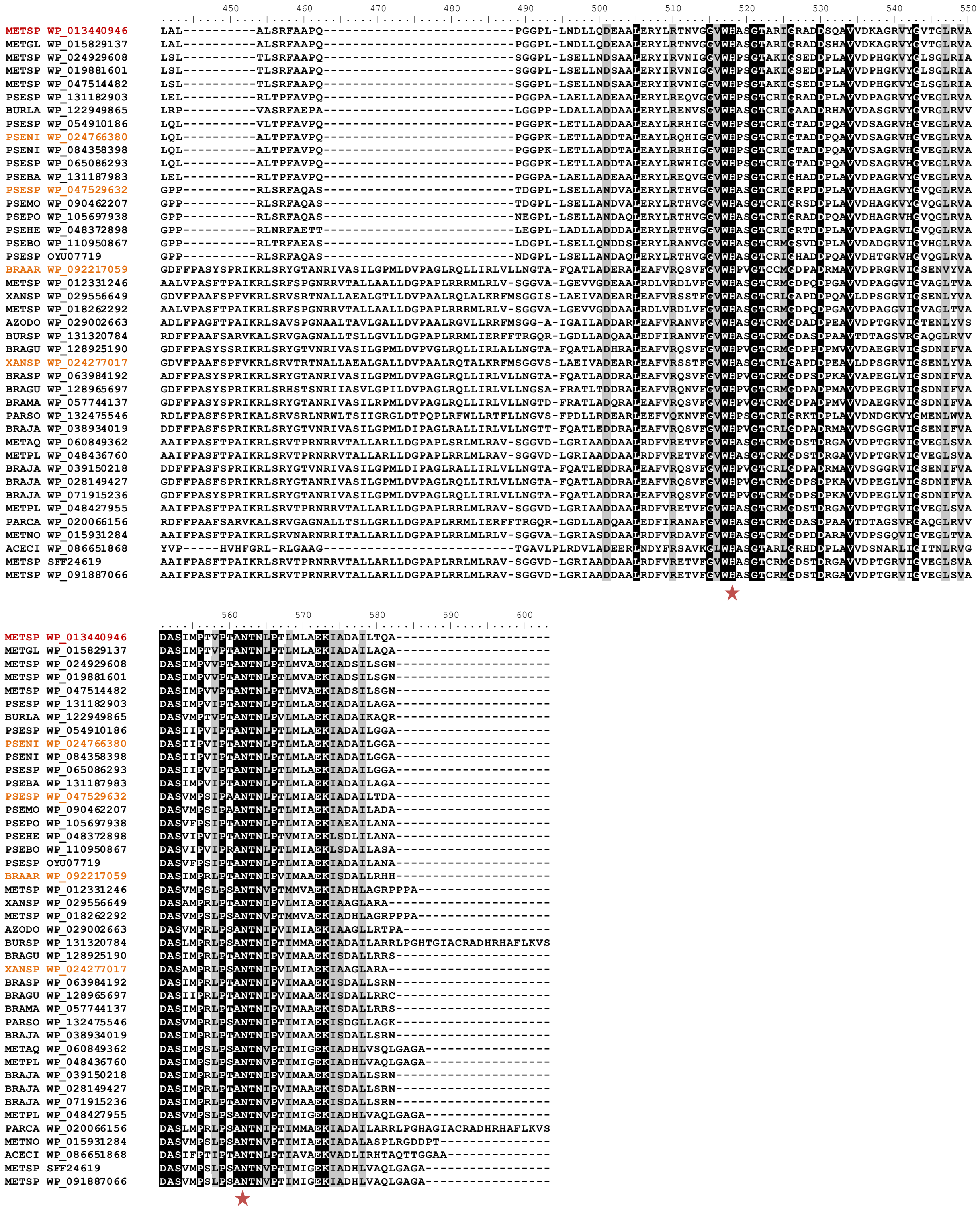

Fig. S1. (cont.)

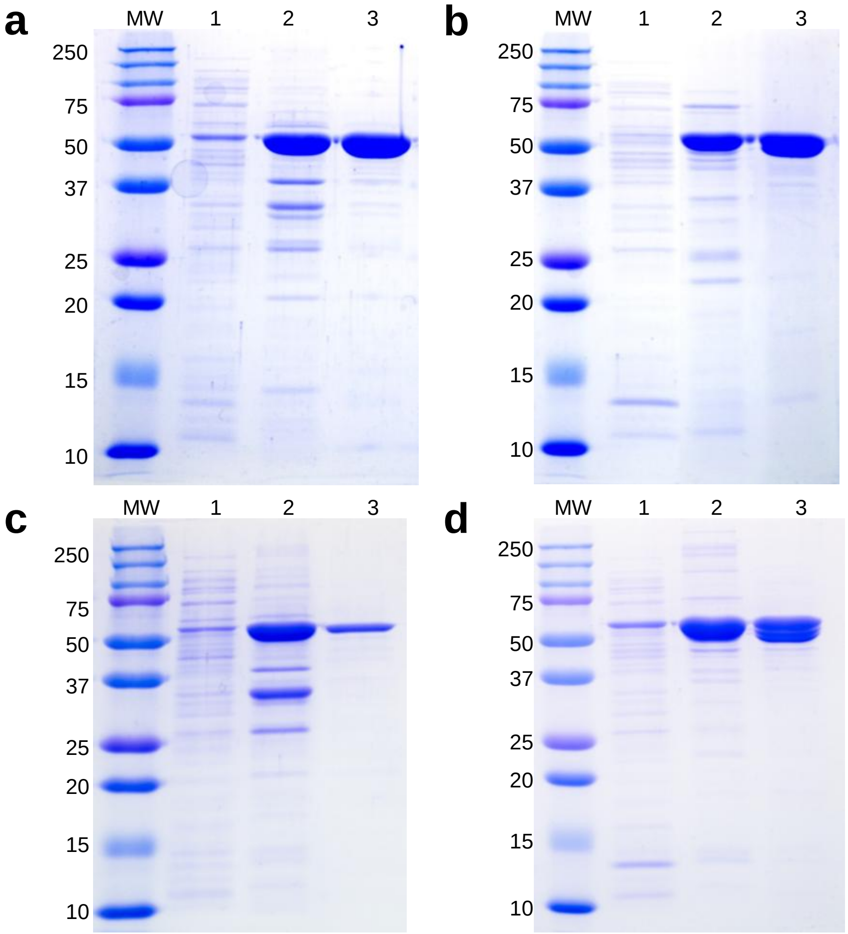

Fig. S2. SDS-PAGE during purification of *Metsp*HMFO (**a**), *Metsp*HMFO^{His} (**b**), *Psesp*HMFO (**c**) and *Pseni*HMFO^{His} (**d**). *Lane 1* corresponds to the initial cell lysates. *Lanes 2* and *3* correspond to the protein fractions after ResourceQ and MonoQ chromatography in **a** and **c**, and after HiTrapIMAC and ResourceQ chromatography steps in **b** and **d**, according to **Table S3**.

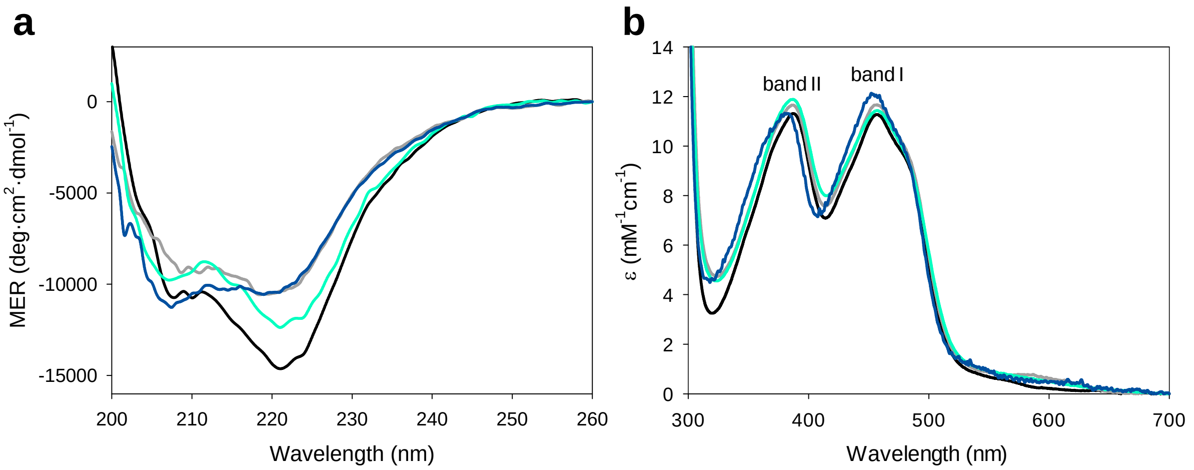

Fig. S3. Spectroscopic analyses of the different HMFOs: Far-UV CD spectra (**a**) and UV-visible spectra (**b**) of *Metsp*HMFO (black), *Metsp*HMFO^{His} (gray), *Psesp*HMFO (cyan) and *Pseni*HMFO^{His} (blue).

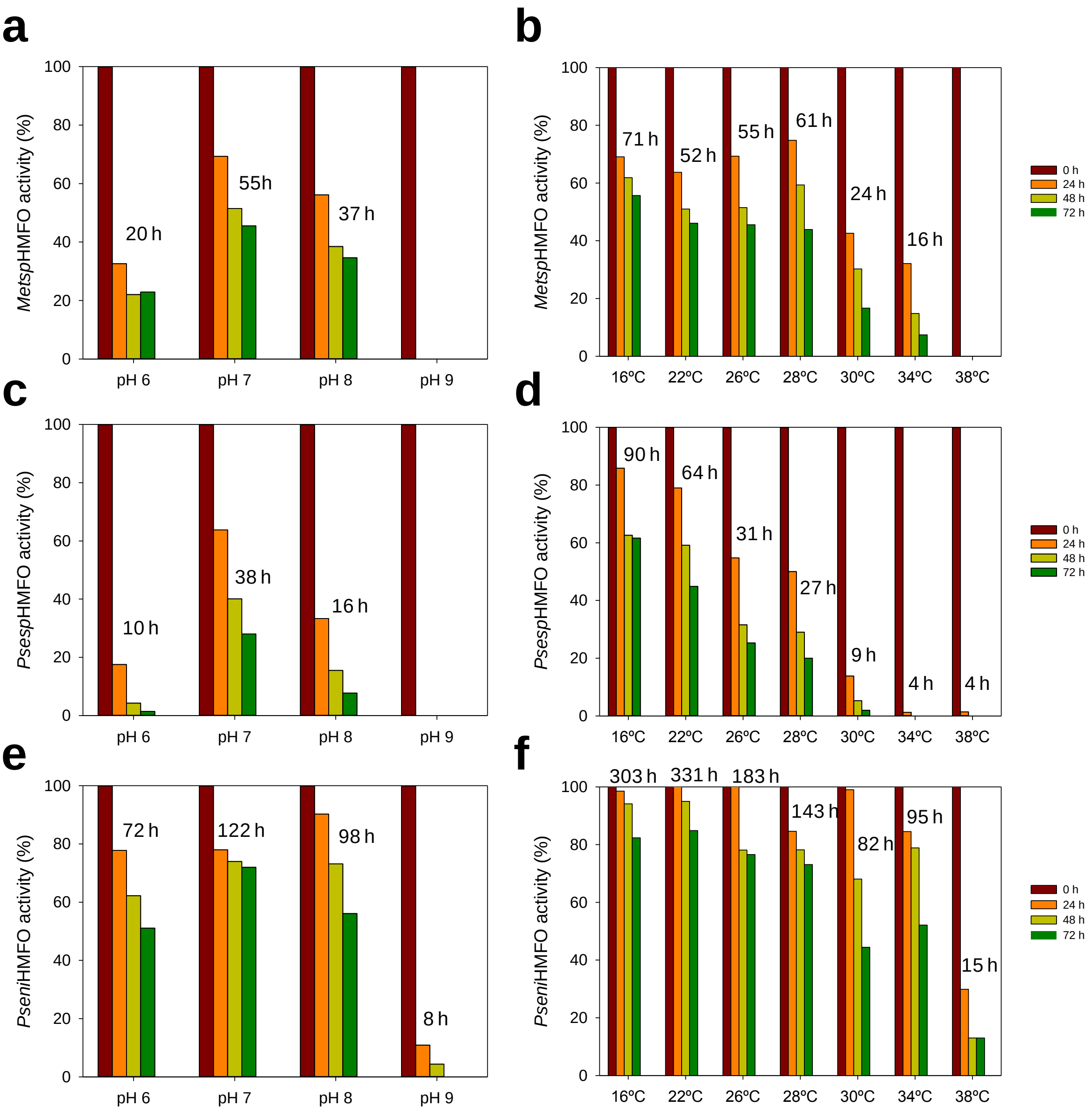

Fig. S4. Residual activity of *Metsp*HMFO (**a** and **b**), *Psesp*HMFO (**c** and **d**) and *Pseni*HMFO (**e** and **f**) during 72-h oxidation of HMF under different pH (**a**, **c** and **e**) and temperature (**b**, **d**, and **f**) conditions. Enzyme half-lifes ($t_{1/2}$) for each condition are indicated on the bars. Activity was measured with vanillyl alcohol in 50 mM Tris/HCl, pH 7.0.

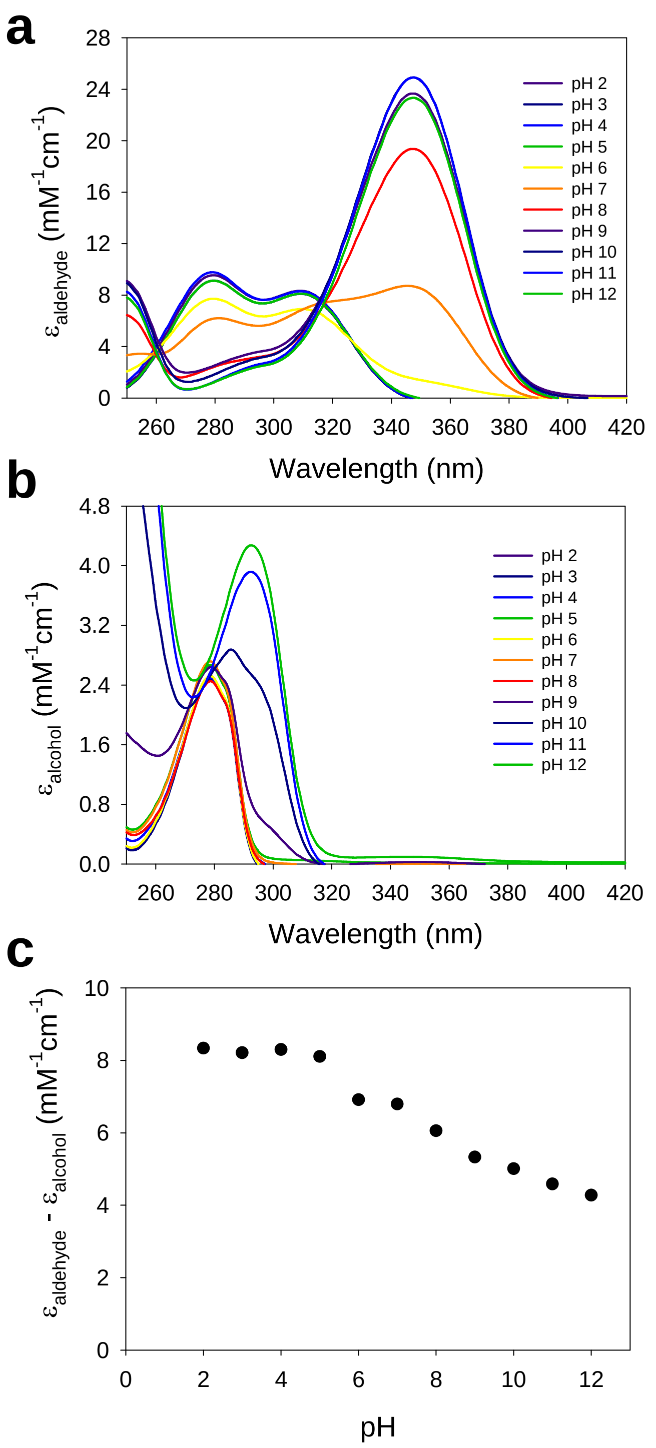

Fig. S5. Effect of pH (2-12 range) on the UV-vis spectra of vanillin (**a**) and vanillyl alcohol (**b**) in 0.1 M B&R buffer, and changes with pH in the difference extinction-coefficient at 309 nm ($\varepsilon_{aldehyde}$ - $\varepsilon_{alcohol}$) used for estimating the vanillyl alcohol transformation into vanillin (**c**).
